# Traditional Chinese medicines and capecitabine‐based chemotherapy for colorectal cancer treatment: A meta‐analysis

**DOI:** 10.1002/cam4.4896

**Published:** 2022-06-01

**Authors:** Hui‐Zhong Jiang, Ya‐Li Jiang, Bing Yang, Feng‐Xi Long, Zhu Yang, Dong‐Xin Tang

**Affiliations:** ^1^ College of Graduate Guizhou University of TCM Guiyang China; ^2^ The First Affiliated Hospital of Guizhou University of TCM Guiyang China

**Keywords:** capecitabine, colorectal cancer, meta‐analysis, traditional Chinese medicine

## Abstract

This meta‐analysis was conducted to evaluate the efficacy and safety of the addition of Traditional Chinese Medicine (TCMs) to capecitabine‐based regimens for colorectal cancer (CRC) in term of tumor. The eight electronic databases including Cochrane Library, PubMed, Web of Science (WOS), Excerpt Medica Database (Embase), Chinese Biomedical Literature Database (CBM), China National Knowledge Infrastructure (CNKI), Chinese Science and Technology Journals (CQVIP), and Wanfang Database were systematically searched for eligible studies from their inception to March 2021. Thirty‐nine randomized controlled trials were involved in this study, and all the data were analyzed by Review Manager 5.3 (Nordic Cochran Centre, Copenhagen, Denmark) and R 4.0.5 software. The meta‐analyses suggested that TCMs in combination with capecitabine‐based regimens increased objective response rate (ORR) in the palliative treatment of CRC (risk ratio [RR], 1.35 [1.17, 1.55], *I* ^2^ = 0%), disease control rate (DCR) (RR, 1.22 [1.12, 1.32], *I* ^2^ = 3%), and quality of life (QOL) (RR, 1.71 [1.44, 2.03], *I* ^2^ = 0%), with decreased risks of myelosuppression, anemia, thrombocytopenia, liver/renal dysfunction, neurotoxicity, nausea/vomiting, neutropenia, diarrhea, leukopenia, improved the peripheral lymphocyte, reduced the expression of tumor markers, and related factors. Further sensitivity analysis of specific plant‐based TCMs found that dangshen, fuling, and gancao had significantly higher contributions to the results of the RR. The results show that capecitabine‐based chemotherapy combined with TCM in the treatment of CRC increases the efficiency of ORR and DCR, reduces chemotherapeutic agents‐associated adverse reactions, and improves their life quality as compared with chemotherapy alone, but further randomized and large sample of studies are needed.

## Graphical abstract

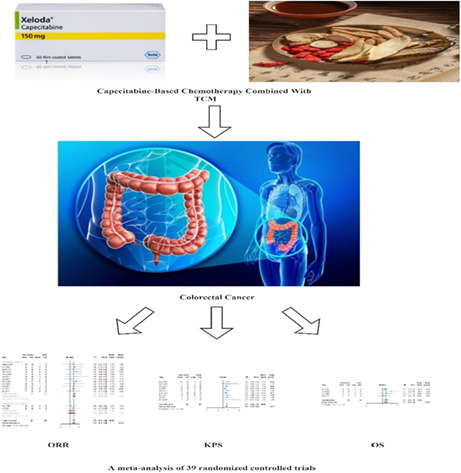

## 1 INTRODUCTION

Globally, the incidence of colorectal cancer (CRC) ranks third among all cancers, second in mortality,^1^ and cancer cases and deaths represent 10% of all cancer cases and deaths.^2^ Despite radiotherapy and chemotherapy is the main treatment method nowadays, the outcome of advanced CRC remains poor due to tumor recurrence and metastasis and drug resistance. For patients who cannot tolerate surgery, the goal is to minimize the tumor and control its further spread and growth.^3^, ^4^

Current first‐line chemotherapy approach to treating CRC is fluoropyrimidine (5‐FU)^5^ or multidrug combination regimen including oxaliplant (OX), irinotecan (IRI), and carbapitabine (CAP). However, the adverse drug reactions during chemotherapy have not been effectively solved, and the treatment outcomes are often unsatisfactory.^6^ As a predrug of fluorouracine, capecitabine achieves similar efficacy after oral administration. The incidence of adverse reactions with the capecitabine modified XELIRI (CAP+ IRI) protocol was significantly reduced.^7^ However, capecitabine still produces adverse reactions such as hand‐foot syndrome, myelosuppression, liver/renal dysfunction, and gastrointestinal reactions during patients.^8^

Traditional Chinese Medicines (TCM) has been widely used in China for the supplementary treatment of cancer, including colorectal cancer (CRC).^9^, ^10^ As an adjuvant therapy, TCM reduces the side effects of cancer reagents and increases the chemotherapeutic efficacy.^11^ However, its substantial evidence is inefficient to prove whether the TCM combined capitabine‐based regimen is more effective than capitabine alone.

In this study a systematic review and meta‐analysis is performed to compare the clinical efficacy and safety between the capecitabine‐based chemotherapy combined with TCM and capecitabine alone in the treatment of CRC. At the same time, the frequencies of combined TCMs are further analyzed to determine which combination methods are efficient to improve objective response rates (ORR) and reduce adverse effects, which will provide evidences for the clinical applications of TCM combinations with capitabine‐based regimen in treating CRC.

## 2 MATERIALS AND METHODS

The protocol for this systematic review was registered on INPLASY (Unique ID number) and was available in full on the inplasy.com (https://doi.org/10.37766/inplasy2021.3.0095) and was performed in accordance with the PreferredReporting Items for Systematic Reviews and Meta‐Analyses (PRISMA) statement.

### 2.1 Eligibility criteria and outcome measures

According to the PICOS acronym,^12^ the inclusion criteria were as follows: Participants (P): All included cases must be confirmed to be CRC after histopathological examination. No restriction on gender, race, or nation was found. Patients with non‐primary CRC or other tumors were excluded.

Intervention (I): The random clinical trials (RCTs) with TCMs combined with capecitabine‐based chemotherapy were included. No restrictions were in the types of TCM.

Comparison (C): In the control groups, the patients with CRC were treated with the capitabine‐based regime.

Outcomes (O): efficacy and safety of TCM.

Study design (S): RCTs.

Exclusion criteria: (i) no capecitabine‐based chemotherapy and (ii) non RCTs and (iii) with incomplete outcomes and (iv) lack in sufficient data. Primary outcomes included three efficacy measurements: short‐ and long‐term clinical efficacy, and adverse drug reactions (ADRs) according to world health organization (WHO) criteria and response evaluation criteria in solid tumors (RECIST). (I) Short‐term clinical efficacy: the short‐term tumor response included complete response (CR), partial response (PR), response rates in stable disease (SD), response rates in progressive disease (PD), ORR, and disease control rate (DCR). ORR was defined as the sum of CR and PR, and DCR was the sum of CR, PR, and SD; (II) Long‐term clinical efficacy: 1–5 year overall survival rate (OS); (III) quality of life (QOL), QOL is considered to be improved when Karnofsky performance status (KPS) score is higher than 10 points after treated. Secondary outcomes included ADRs, peripheral blood lymphocytes, tumor markers and related cytokines, transfer rate, and time to progress (TTP). According to WHO recommendations for grading of acute and subacute toxicity or NCI common terminology criteria for adverse events (CTCAE), ADRs are evaluated by testing hematotoxicity (neutropenia, anemia, thrombocytopenia, and leukopenia), gastrointestinal reaction (nausea and vomiting, diarrhea), liver/renal dysfunction, neurotoxicity, myelosuppression, and hand‐foot syndrome. T‐lymphocyte subsets such as the proportion of CD3^+^, CD4+, and CD8^+^ T cells, the ratio of CD4^+^/CD8^+^ T cells, and the proportion of natural killer cells (NK cells) are measured. Tumor markers and related factors tested include CEA, CA199, CA125, CA724, and TNF‐α.

### 2.2 Search strategy and study selection

Literature search in both international (Cochrane Library, PubMed, EMBASE, and Web of Science) and Chinese (CBM, CNKI, CQVIP, and Wanfang Database) databases will be systematically searched for eligible studies from their inception to March 2021, were independently conducted by two researchers (Hui‐zhong Jiang and Ya‐li Jiang). The retrieved keywords included TCM, CRC, capecitabine, and ADRs. The titles and abstracts were independently screened and then full texts of relevant publications for eligibility were read. Any discrepancy was discussed with a third researcher (Dong‐xin Tang). In addition, the references listed in original reports and previous reviews were reviewed, and manually selected for other available publications.

### 2.3 Data extraction

The following study and participant characteristics were extracted, including first author, year of publication, sample size, type of medications, mean age of participants, cancer staging system (TNM stage), Karnofsky performance status (KPS), TCM intervention (dosage and duration), drug delivery, capecitabine regimen (dose and cycles), and outcome measurements. Any disagreement was resolved by consensus.

### 2.4 Quality assessment and evidence level

The quality of studies were assessed by Cochrane risk of bias tool Review Manager 5.3 (Nordic Cochran aa). The review criteria cover seven areas included random sequence generation, allocation concealment, blinding of participants and personnel, blinding of outcome assessment, incomplete outcome data, selective reporting, other sources of bias. The included studies were evaluated to three degrees including low, unclear, and high risk of bias.

### 2.5 Statistical Analyses

Statistical analyses were performed using Review Manager 5.3 and R 4.0.5 software. The outcomes were mainly represented by risk ratio (RR) and standardized mean difference (SMD) with its 95% CIs. A two‐tailed *p* < 0.05 is considered to be statistically significant. Cochrane's *Q*‐test and *I* ^2^ statistics were used to assess heterogeneity between studies; *p* ≤ 0.1 or *I* ^2^ > 50% indicates statistical heterogeneity. A fixed‐effects model was used to calculate the outcomes when statistical heterogeneity was absent. Otherwise, the random‐effects model was used according to the DerSimonian and Laird method. Studies with zero events were included to avoid overestimation of effect.^13^ When the same outcome was reported by more than 10 studies, publication bias was tested using funnel plots, Egger's regression test, and Begg's rank test. Sensitivity analysis was conducted to explore an individual study's influence on the pooled results by deleting one single study each time from pooled analysis.

Subgroup analyses were carried out based on the methods of the TCM administration. Meanwhile, only the TCMs with significant tumor responses were included in our analyses. Pooled ORRs were calculated for each group of studies that contained the same TCM. The same pairs of TCMs in three or more studies were identified. The pooled RRs were calculated. They were listed in descending order and any significant was highlighted.

## 3 RESULTS

### 3.1 Literature search and study characteristics

**FIGURE 1:**
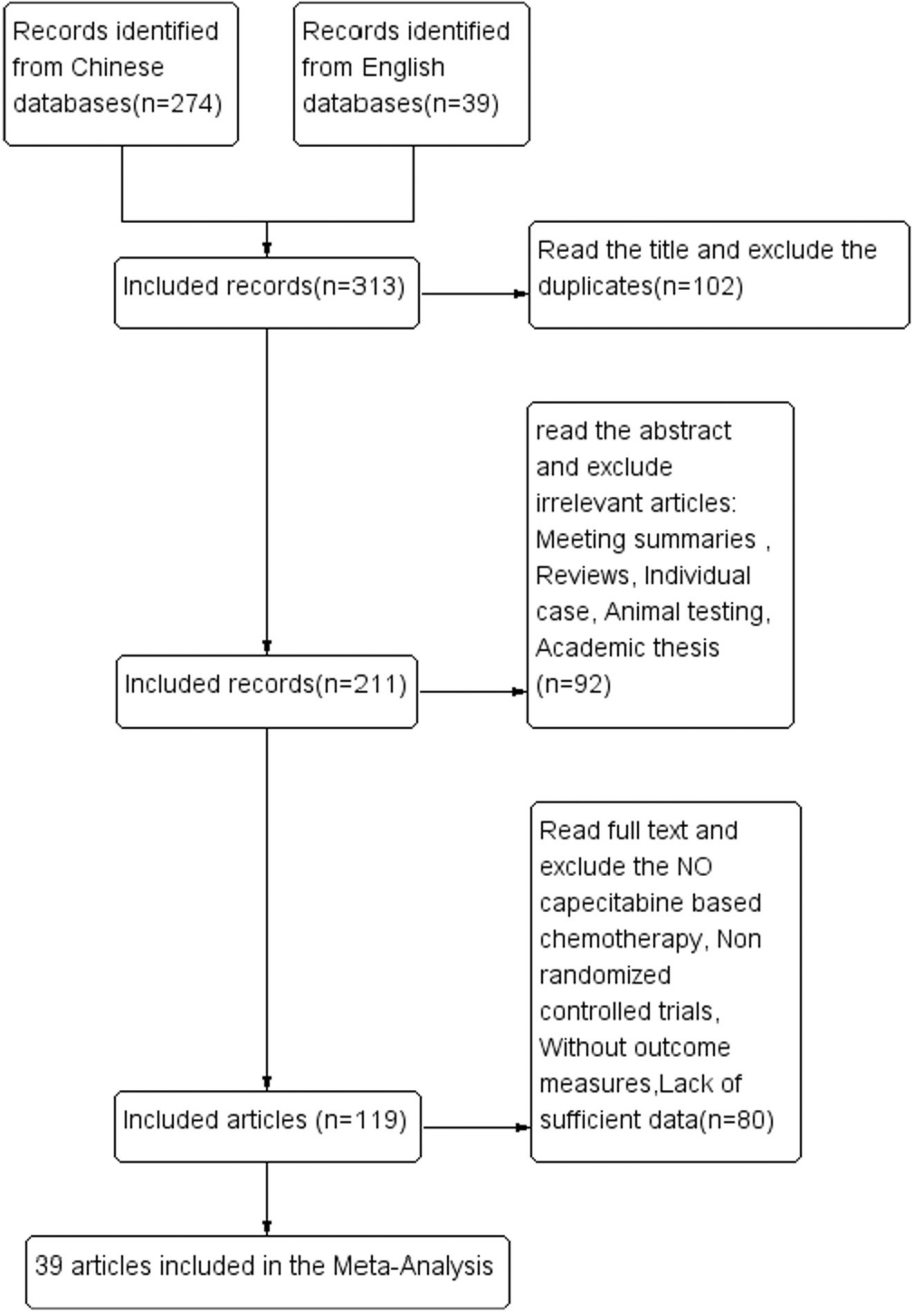
The flow charts of included studies.

A total of 313 articles were initially identified. After screening the titles and abstracts, 119 articles were retrieved for full‐text review. Finally, 39 studies^14^, ^15^, ^16^, ^17^, ^18^, ^19^, ^20^, ^21^, ^22^, ^23^, ^24^, ^25^, ^26^, ^27^, ^28^, ^29^, ^30^, ^31^, ^32^, ^33^, ^34^, ^35^, ^36^, ^37^, ^38^, ^39^, ^40^, ^41^, ^42^, ^43^, ^44^, ^45^, ^46^, ^47^, ^48^, ^49^, ^50^, ^51^, ^52^ with 1384 patients in the TCM combined with capecitabine group and 1367 patients in the capecitabine group were included for meta‐analyses (Figure 1). All the 39 studies were RCTs, and the characteristics are summarized in Table 1. All the 39 studies were conducted in China. Thirty‐five studies used the oral TCM, two studies used external TCM, and two studies used commercially available TCM injections (Table 1).

**TABLE 1:** Characteristics of randomized controlled trials of TCMs combined with capecitabine‐based regiments for CRC

First author (year)	Design	Sample size T/C; Age T/C	TNM (T/C); KPS	TCM intervention; Dosage and duration	Drug delivery	Capecitabine (Cap.) Regimen; Dose, Cycles (T/C)	Outcome measures
Zhao 2017^14^	RCT	30/30; (54.1 ± 4.9)/(53.3 ± 5.6)	II‐III(all); NR	Changningyin, 150 ml, bid, 21 days/cycle, for 2 cycles	Orally	XELOX: Ox. 130 mg/m^2^, ivgtt, d1, capecitabine 1250 mg/m^2^, bid, po, 21 days/cycle (all)	O2
Shi 2018^15^	RCT	48/48; (57.26 ± 7.24)/(59.31 ± 7.97)	II: 7/5, III: 32/35, IV: 9/8; KPS > 60	Erlingyiren decoction, bid, 1 month/cycle, for 3 cycles	Orally	XELOX:Ox. 130 mg/m^2^, ivgtt, d1, capecitabine 1250 mg/m^2^, bid, po, d1‐d14, 21 days/cycle, for 4 cycles (all)	O2, 5
Chen^16^	RCT	48/48; (61.07 ± 8.64)/(60.86 ± 8.57)	II: 40/41, III: 8/7; NR	Erlingyiren decoction, 300 ml, bid, continue for 2 weeks, stop for 1 week, 3 weeks/cycle, for 3 cycles	Orally	XELOX: Ox. 130 mg/m^2^, ivgtt, d1, capecitabine 1000 mg/m^2^, bid, po, d1‐d14, 21 days/cycle, for 3 cycles (all)	O4, 5, 6
Gu^17^	RCT	28/28; (56.5 ± 1.4)/(56.4 ± 1.3)	NR; NR	Erlingyiren decoction, 300 ml, bid, d1‐d14, 3 weeks/cycle	orally	XELOX: Ox. 130 mg/m^2^, ivgtt, day 1, capecitabine 1000 mg/m^2^, bid, po, d1‐d14, 21 days/cycle (all)	O4, 5, 6
Zhang^18^	RCT	30/30; (67 ± 11)/(63 ± 13)	IV (all); KPS ≥70	Jianpijiedufang, 200 ml, bid, 21 days/cycle, for 2 cycles	Orally	Capecitabine 1000 mg/m^2^, bid, po, continue for 2 weeks, stop for 1 week, 3 weeks/cycle, for 2 cycles (all)	O1, 4
Cui^19^	RCT	20/20; 18–70	IV (all); KPS ≥60	Jianpiquyufang, 200 ml, bid, for 8 weeks	Orally	Xeloda+CPT‐11: Xeloda 1000 mg/m^2^, bid, for 14 days, CPT‐11 60 mg/m2, ivgtt for d1, d8, d15, 4 weeks/cycle, for 2 cycles(all)	O2
Sun^20^	RCT	29/29; (54.6 ± 4.8)/(56.0 ± 4.6)	NR;NR	Jianpiziyin decoction, 300 ml, tid, for 14 days	Orally	XELOX: Ox. 130 mg/m^2^, 3 hours ivgtt, capecitabine 1000 mg/m^2^, bid, po, d1‐d14 (all)	O6
Ding^21^	RCT	32/30; (35–76)/(37–78)	III‐IV (all); KPS > 70	Kang'ai injection, 40 ml, ivgtt, qd, d1‐d14, 21 days/cycle	Injection	XELOX: capecitabine 1000 mg/m^2^, bid, 0.5 h p.c. po, d1‐d14, Ox. 130 mg/m^2^, 2 h ivgtt, d1, 21 days/cycle (all)	O1, 2, 4
Min^22^	RCT	41/41; (59.05 ± 6.42)/(59.89 ± 6.71)	III (29), IV (12)/III (30), IV (11)	Shiyiwei Shenqi Capsules, 5 pills, tid	Orally	XELOX: capecitabine 1250 mg/m^2^, bid, d1‐d14, Ox. 130 mg/m^2^, 6 hs ivgtt, d1, 3 weeks/cycle, for 6 cycles (all)	O4, 6
Pan^23^	RCT	32/32; (58.7 ± 3.2)/(59.2 ± 3.3)	II (13), III (19)/II (12), III (20); KPS ≥60	Silingsan, 300 ml, bid, po, 3 weeks/cycle, for 4 cycles	Orally	XELOX: Ox. 130 mg/m^2^, 2 h ivgtt, d1, capecitabine 1000 mg /m^2^, bid, 3 weeks/cycle, for 4 cycles (all)	O2, 5, 6
Yue^24^	RCT	37/39; (51.65 ± 12.15)/(51.86 ± 12.05)	IV (all); KPS ≥60	Tongtai decoction 150 ml, bid	Orally	XELIRI: irinoteca 100/m^2^, ivgtt, d1‐d14, capecitabine, po, bid, 21 days/cycle, for 2 cycles (all)	O1, 2, 4, 6
Zhou^25^	RCT	60/60; (57.9 ± 9.8)/(58.2 ± 9.6)	NR; NR	TCM, bid, 1 h p.c. d1‐d14, 21 days/cycle	Orally	Capecitabine 2000 mg/m^2^, bid, 0.5 h p.c. treatmeat for 2 weeks, stop 1 week (all)	O4, 5
Gu^26^	RCT	60/60; (53.62 ± 6.74)	II (47), III (73); KPS ≥70	Xiaoaiping injection, p.r., qd, 2 weeks/cycle, for 4 weeks	Enema	XELOX: Ox.130 mg/m^2^, 2 h ivgtt, d1, capecitabine 1000 mg/m^2^, po, bid, d1‐d14, 21 days/cycle, for 2 cycles (all)	O2, 4
Xiao^27^	RCT	30/30; 37–74/39–72	NR; KPS ≥60	Guiqiliujun decoction, bid, 21 days/cycle, for 2 cycles	Orally	XELOX: Ox.130 mg /m^2^, 3 h ivgtt, d1, capecitabine 1000 mg /m^2^, po, d1‐d14, 21 days/cycle, for 2 cycles (all)	O1, 2, 3, 5, 8
Bin^28^	RCT	40/36; NR	NR; KPS ≥70	Zhenxiang capsules, 6 pills, tid, 0.5 h p.c.	Orally	Capecitabine 2500 mg/m^2^, po, d1‐d14, 21 days/cycle, for 2 cycles (all)	O5
Yao^29^	RCT	21/21; (62.45 ± 9.64)/(57.5 ± 10.35)	IV (all); KPS > 60	TCM, bid, po, for 6 cycles	Orally	XELOX: Ox.1350 mg/m^2^, d1, 2 h ivgtt, capecitabine 1000 mg/m^2^, po, d1‐d14, 21 days/cycle, for 2 cycles (all)	O2, 4, 6
Guo^30^	RCT	45/45; (55.1 ± 6.1)	III (52), IV (28); KPS > 60	Fuzhengxiaoji decoction, bid or tid, d1, 14 days/cycle, for 3 cycles	Orally	Ox. 85 mg/m^2^, ivgtt, d1, capecitabine 1000 mg/m^2^, po, d1‐d14, 21 days/cycle, for 2 cycles (all)	O1, 5, 6
Chen^31^	RCT	28/28; 34–73	III (32), IV (24); KPS ≥60	Shenyi capsules, 20 mg, bid, 6 weeks/cycle, for 2 cycles	Orally	Capecitabine 2000 mg/m^2^, po, d1‐d14, 0.5 h p.c. Ox. 85 mg/m^2^, 2 h ivgtt, 21 days/cycle, for 4 cycles (all)	O1, 2, 4, 5
Liu^32^	RCT	40/40; (61.28 ± 5.05)/(61.85 ± 4.93)	IV (all); KPS > 60	Shengxuefang, bid, for 6 weeks	Orally	XELOX: Ox.130 mg/m^2^, ivgtt, d1, capecitabine 1000 mg/m^2^, po, bid, d1‐d14, 21 days/cycle (all)	O1, 2, 4, 5
Yao^33^	RCT	45/40; 68–80	NR; KPS ≥70	TCM, bid, 0.5 h p.c. d3, 14 days/cycle	Orally	XELOX: capecitabine 1000 mg/m^2^, po, bid, d1‐d14, Ox. 130 mg/m^2^, ivgtt, d1, 28 days/cycle, for 4 cycles (all)	O2, 3, 4
Li^34^	RCT	30/30; 48–69	NR; NR	TCM, 300 ml, pr, 30 min‐1 h, p.r.	enema	Capecitabine 1.5 g, po, bid, d1‐d14, 21 days/cycle (all)	O1, 2, 4
Zhou^35^	RCT	27/26; (59.19 ± 6.83)/(58.50 ± 7.62)	IV (all); KPS ≥60	TCM, 90 ml, bid, p.c. for 4 cycles	Orally	XELOX: Ox. 130 mg/m^2^, ivgtt, d1, capecitabine 1000 mg/m^2^, bid, d1‐d14, 4 weeks/cycle, for 4 cycles (all)	O2, 5, 6
Jiao^36^	RCT	45/45; 55–78/45–75	IV (all); NR	Zibu decoction, 100 ml, bid	orally	Capecitabine 1250 mg/m^2^, po, bid, d1‐d14, 21 days/cycle (all)	O2, 4
Liu^37^	RCT	23/22; (56 ± 12)/(59 ± 11)	NR; KPS > 90	Fuzheng shengbai orally liquid, 20 ml, tid, for 3 months	Orally	Capecitabine 1250 mg/m^2^, bid, for 14 days, Ox. 130 mg/m^2^, d1, 21 days/cycle, for 4 cycles (all)	O1
Dong^38^	RCT	64/58; (76 ± 5.27)/(57.76 ± 4.38)	NR; KPS ≥60	Fufang tengligen preparation, 500 ml, bid	Orally	Irinoteca 180 mg/m^2^, ivgtt, d1, capecitabine 1000 mg/m^2^, po, bid, d1‐d14, 3 weeks/cycle, for 4 cycles (all)	O1, 2, 4, 5
Sun^39^	RCT	18/18; 38–72	NR; KPS ≥60	Pebenyiyang decoction, 200 ml, po, bid	orally	CapeOx: capecitabine 1250 mg/m^2^, po, bid, d1‐d14, Ox. 130 mg/m^2^, 2 hours ivgtt, 21 days/cycle (all)	O1, 4
Xu^40^	RCT	43/43; (59.24 ± 6.45)/(58.87 ± 7.21)	NR; KPS ≥60	Shengxue decoction, 200 mL, qd, for 6 weeks	Orally	Xelox: Ox. 130 mg/m^2^, ivgtt, d1, capecitabine 1000 mg/m^2^, po, bid, d1‐d14, 21 days/cycles, for 2 cycles (all)	O1, 2, 4, 5
Xu^41^	RCT	50/50; (72.3 ± 5.9)	II (25), III (75); NR	TCM, bid, for 2 cycles	orally	Ox. 130 mg/m^2^, d1, 3 h ivgtt, capecitabine 1000 mg/m^2^, po, bid, d1‐d14, 21 days/cycle, for 2 cycles (all)	O1, 7, 8
Ding^42^	RCT	35/35; (47.5 ± 8.6)/(48.2 ± 7.5)	II (13), III (22)/II (15), III (20); KPS ≥60	Aidi injection 100 ml, ivgtt, qd, d1‐d7, d15‐d21	Injection	XELOX: Ox. 130 mg/m^2^, 3 h ivgtt, d1, capecitabine 1000 mg/m^2^, po, bid, d1‐d14, 21 days/cycle, for 3 cycles (all)	O2, 4
Xie^43^	RCT	32/32; 58.35 ± 1.32	II‐III (all); KPS ≥60	Boerning capsules, 0.6 g, tid, for 4 weeks	Orally	XELOX: Ox. 130 mg/m^2^, 2 hours ivgtt, d1, capecitabine 1000 mg/m^2^, po, bid, d1‐d14, 21 days/cycle, for 4 cycles (all)	O1, 2, 4, 5
Ma^44^	RCT	23/23; (53.28 ± 4.62)/(53.37 ± 4.83)	II (4), III (19); KPS: 76.23 ± 7.93	Buqiyichangfang, bid	Orally	Ox. 130 mg/m^2^, capecitabine 1250 mg/m^2^, bid, d1‐d14, 3 weeks/cycle, for 6 cycles (all)	O5, 6
Bian^45^	RCT	20/20; 59.2	II (15), III (23), IV (2); NR	Gubenyiliufang, qd, for 3 weeks	orally	Capecitabine 1250 mg/m^2^, bid, po, 3 weeks/cycle, for 4 cycles (all)	O2, 5, 6
Li^46^	RCT	40/40; (63.51 ± 5.63)/(62.47 ± 5.71)	II‐IV (all); KPS ≥60	Jianpi Fuzheng Recipe, 300 ml, bid, 3 weeks/cycle, for 2 cycles	Orally	Ox. 130 mg/m^2^, 2 hours ivgtt, d1, capecitabine, 1000 mg/m^2^, po, bid, d1‐d14, 3 weeks/cycle, for 2 cycles (all)	O2, 5
Xie^47^	RCT	30/30; (65.7 ± 3.5)/(65.2 ± 2.8)	NR; NR	Jianpiyiqi, 300 ml, bid	Orally	Capecitabine 2000 mg/m^2^, bid, 0.5 h p.c. d1‐d14, 21 days/cycle (all)	O2, 5
Qi^48^	RCT	30/30; (54.1 ± 5.1)/(54.3 ± 5.8)	II‐III (all); NR	Ningchangyin, po, bid	Orally	XELOX: Ox. 130 mg/m^2^, 2 h ivgtt, d1, capecitabine 1250 mg/m^2^, po, bid, d1‐d14, 21 days/cycle, for 4 cycles (all)	O4
Zhou^49^	RCT	30/30; (53.63 ± 7.78)/(55.53 ± 8.12)	II‐III (all); KPS ≥60	Sanmiao granules, tid, 21 days/cycle, for 3 cycles	Orally	XELOX: capecitabine 1.25 g/m^2^, 14 days/cycle, Ox. 85 mg/m^2^, 2 hours ivgtt, for 3 weeks (all)	O2, 4, 5
Wu^50^	RCT	40/40; (47.52 ± 5.11)/(48.03 ± 6.29)	II (8), III (13), IV (19)/II (7), III (12), IV (21); KPS ≥60	Prunella vulgaris tablets, 6 pills, bid, 3 weeks/cycle, for 2 cycles	orally	XELOX: Ox. 130 mg/m^2^, 2 hours ivgtt, d1, capecitabine 800–1000 mg/m^2^, po, bid, d1‐d14, 21 days/cycle, for 2 cycles (all)	O2, 4, 6
Xiao^51^	RCT	30/30; NR	II‐III (all); NR	Tonifying Qi and nourishing Yin prescription, 150 ml, bid, p.c. for 2 weeks	Orally	XELOX: Ox. 130 mg/m^2^, 2 h ivgtt, d1, capecitabine 800–1000 mg/m^2^, po, bid, d1‐d14, 21 days/cycle, for 2 cycles (all)	O6
Xiao^52^	RCT	30/30; 60–71	II (18), III (42); NR	Yiqi Yangyin Huatan Recipe, 150 ml, bid, 0.5 h p.c.	Orally	XELOX: Ox. 130 mg/m^2^, 2 h ivgtt, d1, capecitabine 800–1000 mg/m^2^, po, bid, d1‐d14, 21 days/cycle, for 2 cycles (all)	O2,5

Abbreviations: bid, twice per day; C, control group; d, day; ID, intravenous drip; ivgtt, injection venosa gutta; KPS, Karnofsky Performance Status; N, number; NR, not reported; O: outcomes, O1: tumor response including the objective response rate (ORR), and disease control rate (DCR); O2: quality of life (QOL), O3: Overall Survival rate (OS); O4: adverse drug reactions (ADRs); O5: the levels of peripheral blood lymphocytes; O6: tumor markers and related factors; O7:transfer rate,O8: time to progress (TTP); Ox., oxaliplatin; p.c., post cibum; po, per os; pr, per rectum; qd, once per day; T, treatment group; TCM, traditional Chinese medicine; tid, thrice per day; TNM, Tumor Node Metastasis (“T” for tumor, denotes the extent of invasion of the intestinal wall, “N” for lymphatic node, the amount of lymphatic node involvement, and “M” for the metastasis); Xel/Cap., capecitabine; XELOX, Ox. + capecitabine; XELIRI, irinoteca + capecitabine.

### 3.2 Methodological bias of the included studies

**FIGURE 2:**
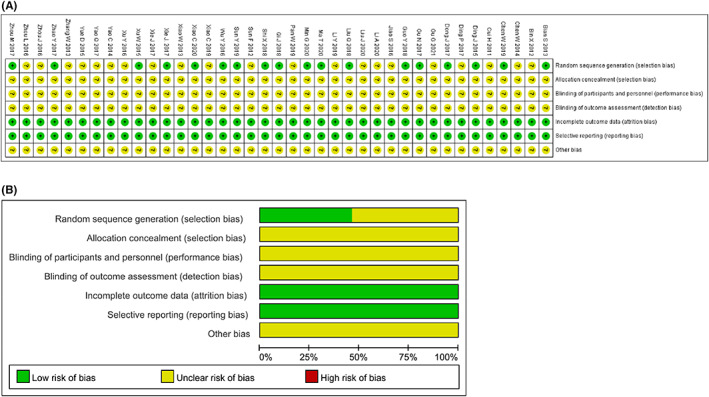
Risk of methodological bias of the included studies. (A) Risk of bias summary: review authors' judgments about each risk of bias item for each included study. (B) Risk of bias graph: review authors' judgment about each risk of bias item presented as percentages across all included studies.

In 39 trials, the methods of random allocation were described clearly in only 18 trials.^14^, ^15^, ^16^, ^22^, ^26^, ^30^, ^37^, ^38^, ^39^, ^40^, ^42^, ^44^, ^45^, ^47^, ^48^, ^49^, ^50^, ^52^ This indicated that there was selectivity bias in the included studies. The random allocation concealment was unclear. Not all the included studies were described as blinding to patients and doctor. Therefore, it indicated that there were selective bias and implementation bias. All data were complete and selective report did not appear in all of the studies. Other bias was not clear. Characteristics and quality of all included studies are presented in Figure 2.

### 3.3 Tumor response

**FIGURE 3:**
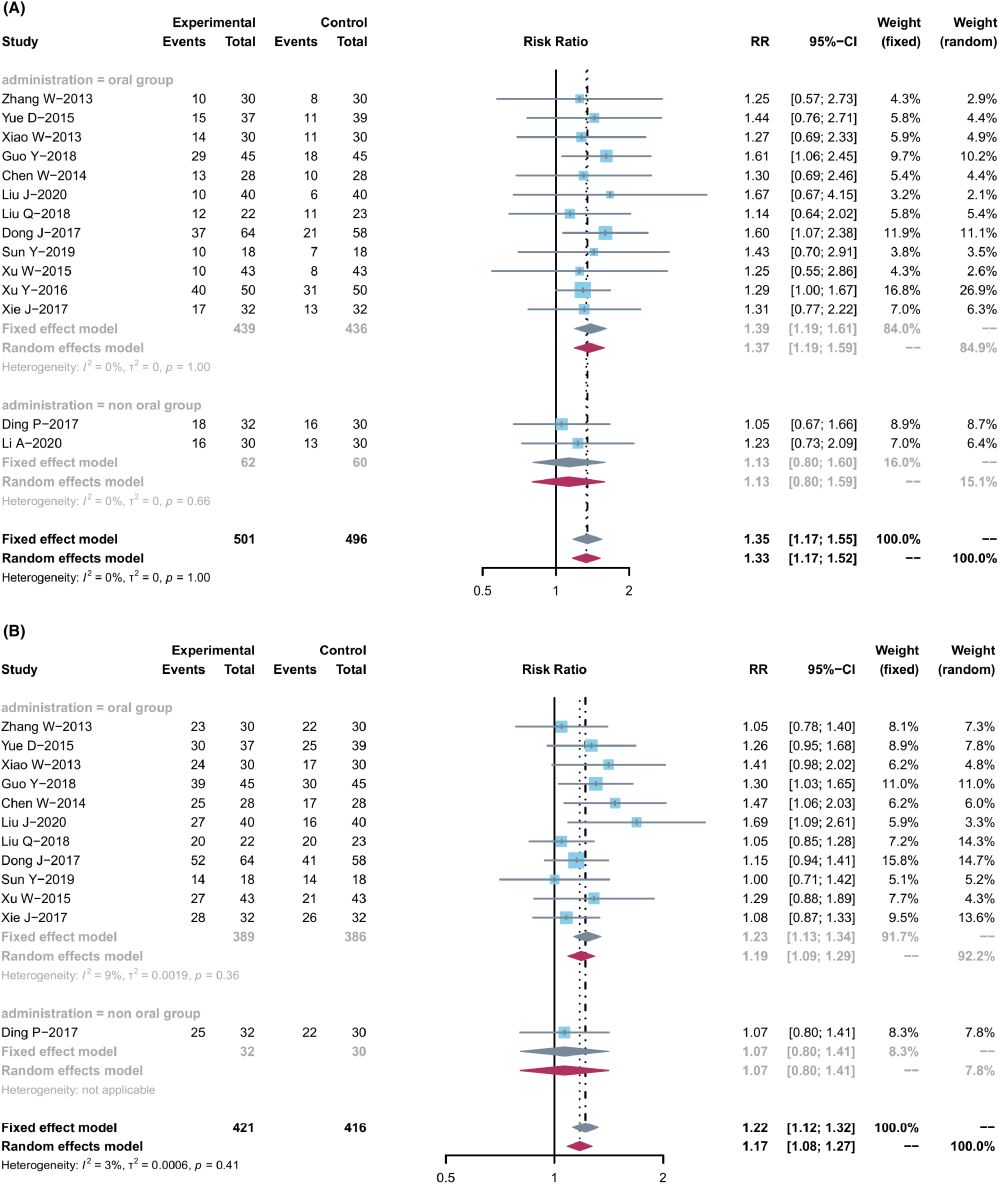
Tumor response. (A) Forest plot displaying the results of the meta‐analysis for ORR. (B) Forest plot displaying the results of the meta‐analysis for DCR.

According to the WHO^53^ or RECIST^54^ guidelines, 14 trials^18^, ^21^, ^24^, ^27^, ^30^, ^31^, ^32^, ^34^, ^37^, ^38^, ^39^, ^40^, ^41^, ^43^ containing 997 and 12^18^, ^21^, ^24^, ^27^, ^30^, ^31^, ^32^, ^37^, ^38^, ^39^, ^40^, ^43^ trials containing 837 cases evaluated ORR and DCR, respectively (Figure 3A,B). Cochran's *χ* ^2^ test and *I* ^2^ statistic showed no heterogeneity (ORR, *I* ^2^ = 0%; DCR, *I* ^2^ = 3%). Therefore, the data both using an FEM were synthesized. Compared with capecitabine alone, TCMs in combination with capecitabine significantly increased ORR (RR, 1.35 [1.45–1.88], *p* < 0.00001) and DCR (RR, 1.22 [1.12, 1.32], *p* < 0.00001).

Two groups were divided for meta‐analyses to evaluate ORR: non‐oral group (2 studies) and oral group (12 studies). The non‐oral group has different ways (e.g., Kang'ai injection, and enema TCM) were tested in two studies (*n* = 122). Significant improvement in ORR (RR, 1.13 [0.80, 1.60], *I* ^2^ = 0%) was found in the non‐oral group. Twelve studies (*n* = 875) were included in the oral group, including decoctions, capsules, or tablets. The pooled ORR showed significant improvement in the oral group (RR, 1.39 [1.19, 1.61], *I* ^2^ = 0%).

One study and 11 studies were included to evaluate the DCR in the non‐oral and oral groups. Similarly, compared with capecitabine alone, the combined treatment significantly improved the pooled DCR in the non‐oral and oral groups (*n* = 62, RR, 1.07 [0.80, 1.41]; *n* = 775, RR, 1.23 [1.13, 1.34], *I* ^2^ = 9%), respectively.

### 3.4 Quality of life

**FIGURE 4:**
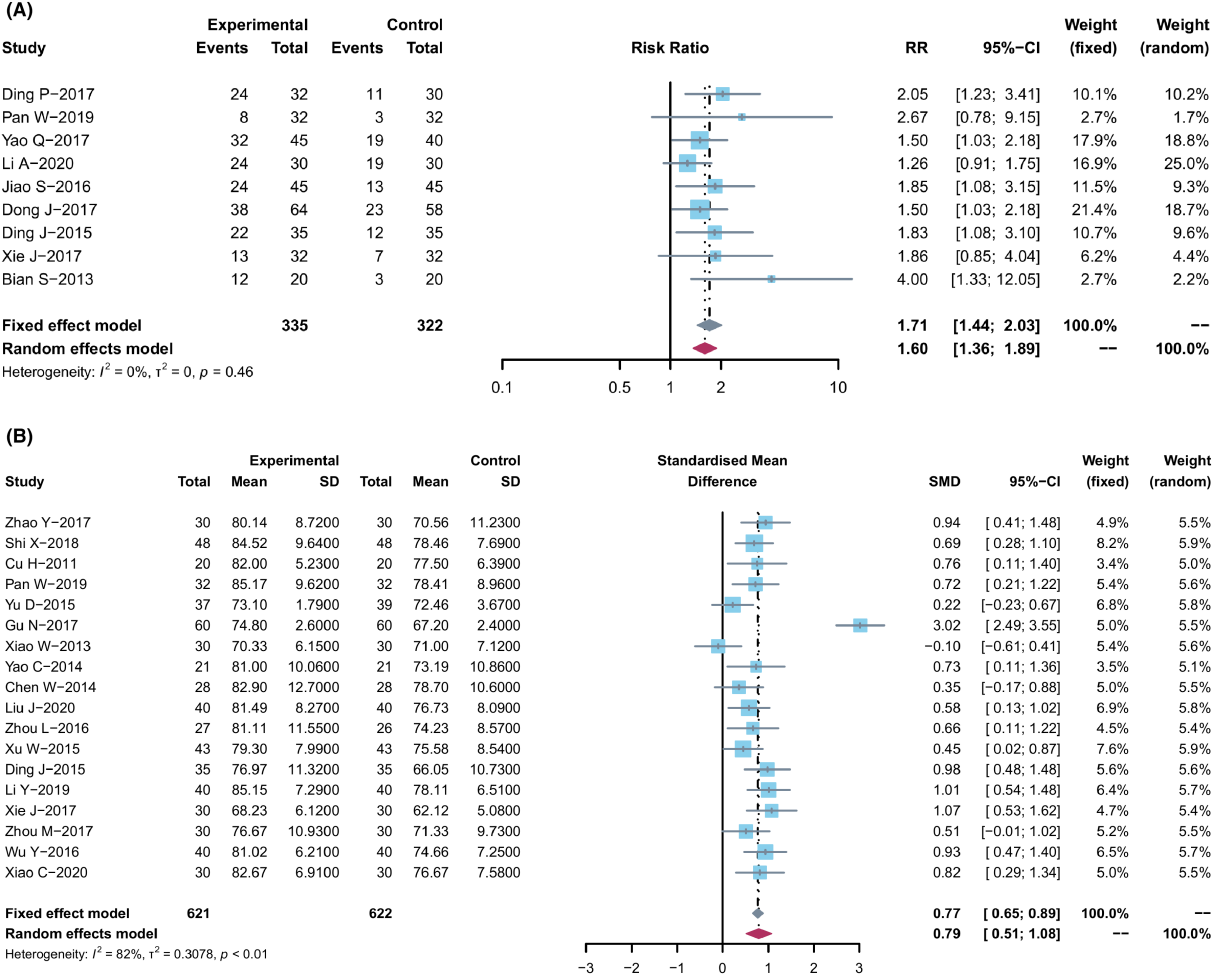
Quality of life. (A) Forest plot displaying the results of the meta‐analysis for KPS according to number of patients. (B) Forest plot displaying the results of the meta‐analysis for KPS according to mean ± SD.

The quality of life (QOL) changes on KPS were reported as two types of data in the included studies, the number of patients^21^, ^23^, ^33^, ^34^, ^36^, ^38^, ^42^, ^43^, ^45^ who reported the improved or stable performance status based on KPS (10‐point cutoff) and the mean ± SD of KPS before and after treatment.^14^, ^15^, ^19^, ^23^, ^24^, ^26^, ^27^, ^29^, ^31^, ^32^, ^35^, ^40^, ^42^, ^46^, ^47^, ^49^, ^50^, ^52^ The results showed that compared with capitabine alone, the combined treatment significantly increased the number of improved patients based on KPS (RR, 1.71 [1.44, 2.03]; *p* < 0.0001, *I* ^2^ = 0%) (Figure 4A), and elevated KPS (SMD, 0.79 [0.51, 1.08]; *p* < 0.0001, *I* ^2^ = 82%) (Figure 4B). Taken together, the KPS in TCM combined with capecitabine group was significantly improved compared with the control group.

### 3.5 Overall survival rate

**FIGURE 5:**
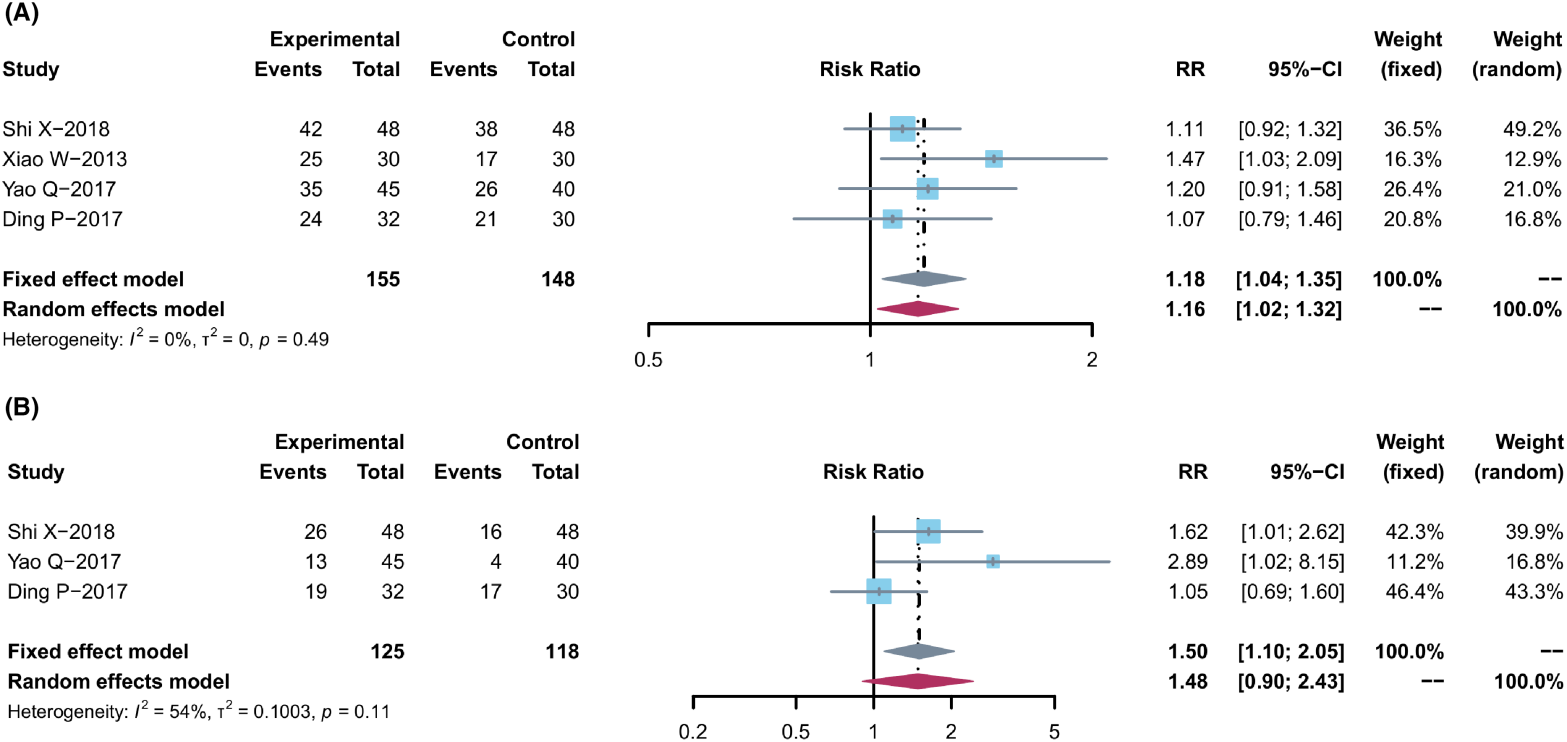
Overall survival rate. (A) Forest plot displaying the results of the meta‐analysis for 1‐year survival rate. (B) Forest plot displaying the results of the meta‐analysis for 2‐year survival rate.

Four trials^15^, ^21^, ^27^, ^33^ with 303 patients reported the 1‐year survival rate. The meta‐analysis showed significant difference between these two treatment groups (RR, 1.18 [1.04, 1.35]; *p* = 0.0126, *I* ^2^ = 0%; Figure 5A). Three trials^15^, ^21^, ^33^ reported the 2‐year survival rate and indicated no statistically significant difference between the two treatment groups (RR, 1.48 [0.90, 2.43]; *p* = 0.1205, *I* ^2^ = 54%) (Figure 5B). Heterogeneity was present after one study (Ding, p. 2017)^21^ was removed (RR, 1.89 [1.22, 2.94], *p* = 0.0047, *I* ^2^ = 3%). These results showed that TCM combined with capecitabine improved 1‐year/2‐year survival rate of CRC patients as compared with capitabine alone.

### 3.6 Adverse drug reactions

Twenty‐one trials^16^, ^17^, ^18^, ^21^, ^22^, ^24^, ^25^, ^26^, ^29^, ^31^, ^32^, ^33^, ^34^, ^36^, ^38^, ^39^, ^40^, ^42^, ^43^, ^48^, ^49^, ^50^ with 1663 cases reported the ADRs (Table 2; Figure S1).Some studies described gastrointestinal reactions and hematological toxicity, but did not distinguish them in detail. Significant heterogeneity in gastrointestinal reaction (*I* ^2^ = 90%), nausea/vomiting (*I* ^2^ = 58%), hand‐foot syndrome (*I* ^2^ = 93%), and hematological toxicity (*I* ^2^ = 97%). The results also showed that TCM combined with capecitabine‐based chemotherapy had lower risks of neutropenia (RR, 0.67 [0.54, 0.85], *p* = 0.0006), thrombocytopenia (RR, 0.76 [0.58, 0.99], *p* = 0.0409), leukopenia (RR, 0.70 [0.60, 0.82], *p* < 0.0001), nausea/vomiting (RR, 0.67 [0.50, 0.88], *p* = 0.0049), diarrhea (RR, 0.61 [0.49, 0.74], *p* < 0.0001), liver/renal dysfunction (RR, 0.64 [0.47, 0.86], *p* = 0.0025), myelosuppression (RR, 0.67 [0.54, 0.82], *p* < 0.0001), anemia (RR, 0.69 [0.52, 0.92], *p* = 0.012), neurotoxicity (RR, 0.79 [0.64, 0.98], *p* = 0.0344) than those of chemotherapy alone. There were no significant differences in RR values and their 95% CI of gastrointestinal reaction (RR, 0.75 [0.50, 1.14], *p* = 0.18), hand‐foot syndrome (RR, 0.62 [0.23, 1.67], *p* = 0.3449), hematological toxicity (RR, 0.62 [0.09, 4.26], *p* = 0.6244) between the two groups.

**TABLE 2:** Meta‐analysis results of ADRs

Outcomes	Trials	Experimental group (Events/Total)	Control l group (Events/Total)	SM	RR,95% CI	*I* ^2^ (%)	*p*	PB
Myelosuppression	9	81/302	121/300	FEM	0.67 (0.54, 0.82)	0	<0.0001	No
Gastrointestinal reaction	8	81/288	121/286	REM	0.75 (0.50, 1.14)	90	0.18	Unclear
Anemia	5	49/217	71/213	FEM	0.69 (0.52, 0.92)	0	0.012	Unclear
Thrombocytopenia	10	70/431	92/428	FEM	0.76 (0.58, 0.99)	0	0.0409	No
Liver/Renal dysfunction	11	52/446	83/442	REM	0.64 (0.47, 0.86)	0	0.0025	No
Neurotoxicity	13	94/432	119/429	FEM	0.79 (0.64, 0.98)	0	0.0344	No
Nausea/vomiting	12	111/447	169/443	REM	0.67 (0.50, 0.88)	58	0.0049	Yes
Neutropenia	3	53/139	77/133	FEM	0.67 (0.54, 0.85)	25	0.0006	Unclear
Hand‐foot syndrome	8	64/281	90/276	REM	0.62 (0.23, 1.67)	93	0.3449	Unclear
Diarrhea	12	93/452	151/443	FEM	0.61 (0.49, 0.74)	0	<0.0001	No
Leukopenia	11	133/435	189/432	FEM	0.70 (0.60, 0.82)	32	<0.0001	Yes
Hematological toxicity	3	40/88	53/88	REM	0.62 (0.09, 4.26)	97	0.6244	Unclear

*Note*: Forest of all results are in Figure S1.

Abbreviations: CI, confidence interval; FEM, fixed‐effects model; PB, Publication bias; REM, random‐effects model; RR, relative ratio; SM, statistical method.

### 3.7 The levels of peripheral blood lymphocytes

Twenty trials^15^, ^16^, ^17^, ^23^, ^25^, ^27^, ^30^, ^31^, ^32^, ^35^, ^38^, ^40^, ^43^, ^44^, ^45^, ^46^, ^47^, ^49^, ^52^ with 1465 cases reported the levels of peripheral blood lymphocytes (Table 3; Figure S2). There was statistical heterogeneity in CD3^+^ T cells (*I* ^2^ = 93%), CD4^+^ T cells (*I* ^2^ = 91%), CD8^+^ T cells (*I* ^2^ = 97%), CD4^+^/CD8^+^ T cells ratio (*I* ^2^ = 92%), and excluded medium heterogeneity in NK cells (*I* ^2^ = 45%). Therefore, the data of CD3^+^ T cells, CD4^+^ T cells, CD8^+^ T cells, and CD4^+^/CD8^+^ T cells ratio and the NK cells were calculated by using a FEM. The meta‐analysis results showed that TCM plus capecitabine‐based chemotherapy improved the CD3^+^ T cells (RR, 1.47 [0.96, 1.98], *p* < 0.0001), CD4^+^ T cells (RR, 1.70 [1.27, 2.13], *p* < 0.0001), CD4^+^/CD8^+^ T cells ratio (RR, 1.47 [1.05, 1.89], *p* < 0.0001), and NK cells (RR, 0.87 [0.69, 1.06], *p* < 0.0001) compared with those of chemotherapy alone. No significant differences were found in RR values and their 95% CI of CD8^+^ T cells (RR, −0.22 [−0.99, 0.54], *p* = 0.565) between the two groups.

**TABLE 3:** Meta‐analysis results of the levels of peripheral blood lymphocytes

Outcomes	Trials	SM	SMD, 95% CI	*I* ^2^ (%)	*p*	PB
CD3^+^ T cells	16	REM	1.47 (0.96, 1.98)	93	<0.0001	Yes
CD4^+^ T cells	17	REM	1.70 (1.27, 2.13)	91	<0.0001	Yes
CD8^+^ T cells	15	REM	−0.22 (−0.99, 0.54)	97	0.565	No
CD4^+^/CD8^+^ T cells ratio	19	REM	1.47 (1.05, 1.89)	92	<0.0001	Yes
NK cells	7	FEM	0.87 (0.69, 1.06)	45	<0.0001	Unclear

*Note*: Forest of all results are in Figure S2.

Abbreviations: CI, confidence interval; FEM, fixed‐effects model; PB, Publication bias; REM, random‐effects model; SM, statistical method; SMD, standardized mean difference.

### 3.8 Tumor markers and related factors

Thirteen trials^15^, ^16^, ^17^, ^23^, ^25^, ^27^, ^28^, ^30^, ^31^, ^32^, ^35^, ^38^, ^40^, ^43^, ^44^, ^45^, ^46^, ^47^, ^49^, ^52^ with 843 cases reported the tumor markers and related factors (Table 4 and Figure S3). In the studies, result showed that there was a significant difference in the level of CEA, CA199, and CA125 between the two groups, and the TCM with chemotherapy group was found to have lower CEA, CA199, and CA125 (RR, −1.83 [−2.69, −0.96], *I* ^2^ = 96%, *p* < 0.0001; −0.86 [−1.32, −0.40], *I* ^2^ = 81%, *p* = 0.0003; −1.73 [−3.14, −0.32], *I* ^2^ = 96%, *p* = 0.0162). But about CA724 and TNF‐α, the result indicated no statistical differences between the two groups (RR, −2.39 [−7.14, 2.36], *I* ^2^ = 99%, *p* = 0.3246; RR, 0.13 [−2.65, 2.91], *I* ^2^ = 98%, *p* = 0.9262).

**TABLE 4:** Meta‐analysis results of tumor markers and related factors

Outcomes	Trials	SM	SMD, 95% CI	*I* ^2^ (%)	*p*	PB
CEA	12	REM	−1.83 (−2.69, −0.96)	96	<0.0001	Yes
CA199	7	REM	−0.86 (−1.32, −0.40)	81	0.0003	Unclear
CA125	4	REM	−1.73 (−3.14, −0.32)	96	0.0162	Unclear
CA724	2	REM	−2.39 (−7.14, 2.36)	99	0.3246	Unclear
TNF‐α	2	REM	0.13 (−2.65, 2.91)	98	0.9262	Unclear

*Note*: Forest of all results are in Figure S3.

Abbreviations: CI, confidence interval; FEM, fixed‐effects model; PB, Publication bias; REM, random‐effects model; SM, statistical method; SMD, standardized mean difference.

### 3.9 Transfer rate and TTP

**FIGURE 6:**
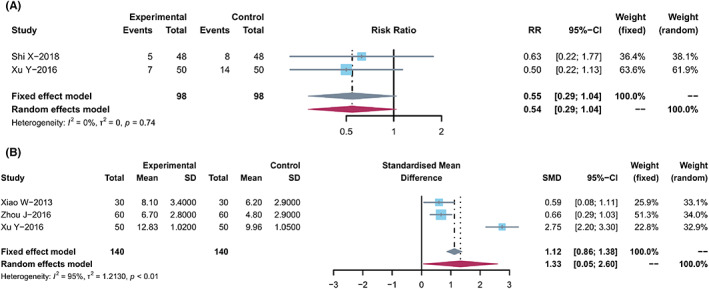
Transfer rate and TTP. (A) Forest plot displaying the results of the meta‐analysis for transfer rate. (B) Forest plot displaying the results of the meta‐analysis for TTP.

Of the 39 trials, only two studies^15^, ^41^reported the tumor transfer rate, and no significant difference was found between the two groups(RR, 0.55 [0.29, 1.03], *I* ^2^ = 0%, *p* = 0.0647) (Figure 6A). And three trials^25^, ^27^, ^41^ reported TTP (RR, 1.33 [0.05, 2.60], *I* ^2^ = 95%, *p* = 0.0419), with a significant difference between the two groups (Figure 6B).

### 3.10 Publication bias analysis

**FIGURE 7:**
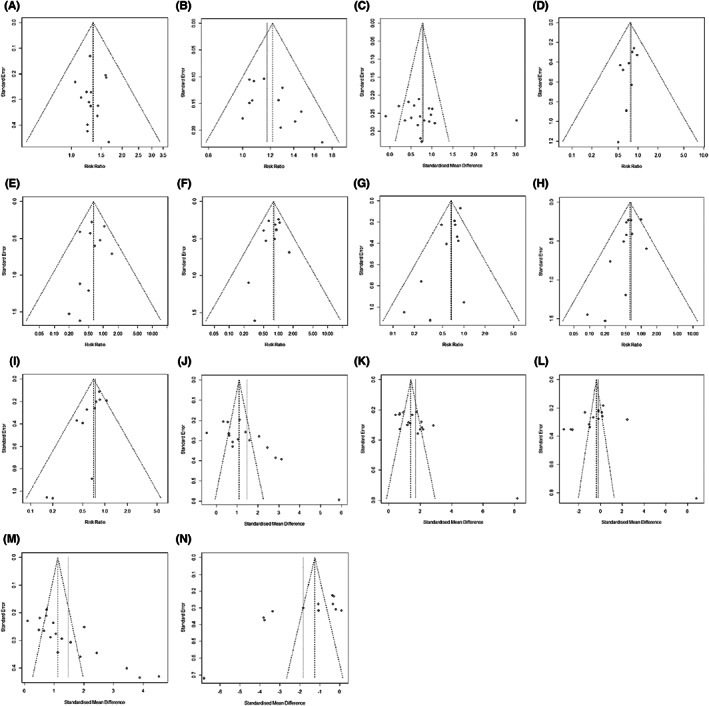
Funnel plots displaying the results of the meta‐analysis for Publication bias analysis. (A) ORR. (B) DCR. (C) KPS (mean ± SD). (D) Thrombocytopenia. (E) Liver/Renal dysfunction. (F) Neurotoxicity. (G) Nausea/Vomiting. (H) Diarrhea. (I) Leukopenia. (J) CD3^+^ T cells. (K) CD4^+^ T cells. (L) CD8^+^ T cells. (M) CD4^+^/CD8^+^ T cells. (N) CEA.

**FIGURE 8:**
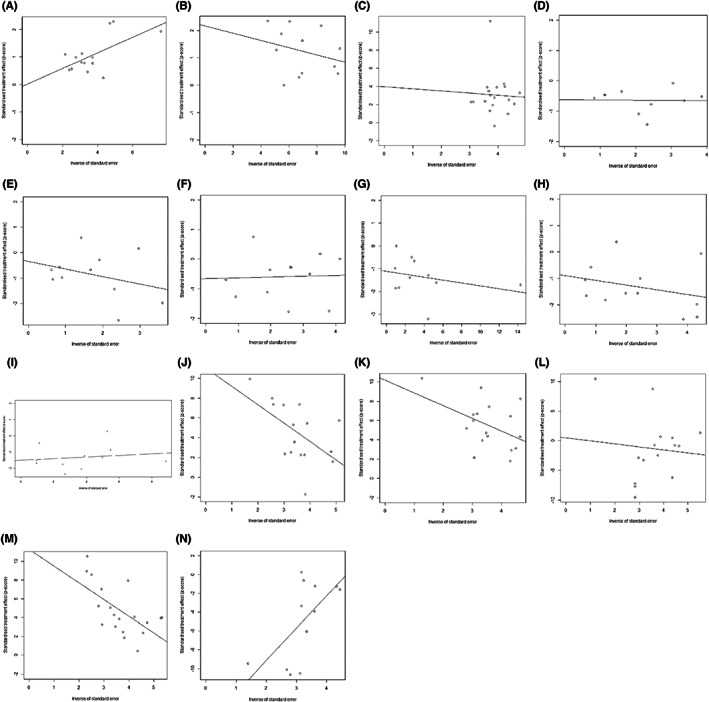
Egger's analysis for Publication bias analysis. (A) ORR. (B) DCR. (C) KPS (mean ± SD). (D) Thrombocytopenia. (E) Liver/Renal dysfunction. (F) Neurotoxicity. (G) Nausea/Vomiting. (H) Diarrhea. (I) Leukopenia. (J) CD3^+^ T cells. (K) CD4^+^ T cells. (L) CD8^+^ T cells. (M) CD4^+^/CD8^+^ T cells. (N) CEA.

More than 10 studies reported the same outcomes, including ORR, DCR, KPS, thrombocytopenia, liver/renaldysfunction, neurotoxicity, nausea/vomiting, diarrhea, leukopenia, CD3^+^ T cells, CD4^+^ T cells, CD8^+^ T cells, CD4^+^/CD8^+^ T cells ratio, and CEA. Publication bias was tested using funnel plots (Figure 7) and Egger's regression test (Figure 8). Other publication bias is shown in the previous tables (Tables 2 and Table 3).

### 3.11 Sensitivity analysis

Thirty‐nine trials were included for sensitivity analysis, excluding the poor/over/ underestimated trials. The result demonstrated that except for CA724, TNF‐α, and transfer rate, no heterogeneities were found in other parameters tested (Table 5 and Figures S4 and S5). Although excluding the trials,^16^, ^17^, ^22^, ^24^, ^29^, ^30^, ^35^, ^45^, ^50^, ^51^ heterogeneity was also found in CEA (Table 5).

**TABLE 5:** Sensitivity analysis by excluding the poor/over/underestimated trials

Outcomes	Trials	SM	RR/SMD, 95% CI	*I* ^2^ (%)	Excluded trials (Reference number)	Trials	SM	RR/SMD, 95% CI	*I* ^2^ (%)
(a)									
ORR	14	FEM	1.35 (1.17, 1.55)	0	1^39^	13	FEM	1.31 (1.13, 1.52)	0
DCR	12	FEM	1.22 (1.12, 1.32)	3	1^32^	11	FEM	1.19 (1.09, 1.29)	0
KPS(number)	9	FEM	1.71 (1.44, 2.03)	0	1^34^	8	FEM	1.80 (1.49, 2.18)	0
KPS(mean ± SD)	18	REM	0.79 (0.51, 1.08)	82	1^26^	17	REM	0.65 (0.53, 0.77)	33
1‐year Survival rate	4	FEM	1.18 (1.04, 1.35)	0	1^27^	3	FEM	1.13 (0.98, 1.30)	0
2‐year Survival rate	3	REM	1.48 (0.90, 2.43)	54	1^21^	2	FEM	1.89 (1.22, 2.94)	3
b)									
Myelosuppression	9	FEM	0.67 (0.54, 0.82)	0	1^21^	8	FEM	0.72 (0.58, 0.88)	0
Gastrointestinal reaction	8	REM	0.75 (0.50, 1.14)	90	1^31^	7	FEM	0.71 (0.59, 0.85)	32
Anemia	5	FEM	0.69 (0.52, 0.92)	0	1^38^	4	FEM	0.76 (0.57, 1.03)	0
Thrombocytopenia	10	FEM	0.76 (0.58, 0.99)	0	1^24^	9	FEM	0.72 (0.54, 0.97)	0
Liver/Renal dysfunction	11	REM	0.64 (0.47, 0.86)	0	1^24^	10	FEM	0.56 (0.40, 0.79)	0
Neurotoxicity	13	FEM	0.79 (0.64, 0.98)	0	1^25^	12	FEM	0.84 (0.67, 1.05)	0
Nausea/Vomiting	12	REM	0.67 (0.50, 0.88)	58	1^15^	11	FEM	0.70 (0.59, 0.84)	41
Neutropenia	3	FEM	0.67 (0.54, 0.85)	25	1^40^	2	FEM	0.56 (0.37, 0.85)	0
Hand‐foot Syndrome	8	REM	0.62 (0.23, 1.67)	93	1^16^	7	FEM	0.58 (0.40, 0.82)	0
Diarrhea	12	FEM	0.61 (0.49, 0.74)	0	4^16^, ^29^, ^38^, ^44^	8	FEM	0.55 (0.43, 0.70)	0
Leukopenia	11	FEM	0.70 (0.60, 0.82)	32	1^33^	10	FEM	0.73 (0.63, 0.86)	18
Hematological toxicity	3	REM	0.62 (0.09, 4.26)	97	1^31^	2	FEM	0.48 (0.27, 0.86)	0
c)									
CD3^+^ T cells	16	REM	1.47 (0.96, 1.98)	93	12^15^, ^16^, ^17^, ^23^, ^31^, ^43^, ^44^, ^47^, ^49^, ^52^	4	FEM	1.26 (1.01, 1.50)	0
CD4^+^ T cells	17	REM	1.70 (1.27, 2.13)	91	8^16^, ^28^, ^30^, ^32^, ^40^, ^43^, ^45^, ^47^	9	FEM	1.74 (1.56, 1.93)	32
CD8^+^ T cells	15	REM	−0.22 (−0.99, 0.54)	97	8^15^, ^23^, ^30^, ^44^, ^45^, ^46^, ^47^, ^52^	7	FEM	−0.06 (−0.23, 0.11)	40
CD4^+^/CD8^+^ T cells ratio	19	REM	1.47 (1.05, 1.89)	92	13^15^, ^16^, ^23^, ^25^, ^27^, ^28^, ^35^, ^38^, ^40^, ^43^, ^46^, ^49^, ^52^	6	FEM	1.24 (1.01, 1.48)	17
NK cells	7	FEM	0.87 (0.69, 1.06)	45	1^49^	6	FEM	0.80 (0.61, 0.99)	5
d)									
CEA	12	REM	−1.83 (−2.69, −0.96)	96	10^16^, ^17^, ^22^, ^24^, ^29^, ^30^, ^35^, ^45^, ^50^, ^51^	2	REM	−1.45 (−2.19, −0.71)	67
CA199	7	REM	−0.86 (−1.32, −0.40)	81	3^17^, ^44^, ^45^	4	FEM	−0.63 (−0.86, −0.39)	48
CA125	4	REM	−1.73 (−3.14, −0.32)	96	2^22^, ^35^	2	FEM	−1.23 (−1.58, −0.88)	45
CA724	2	REM	−2.39 (−7.14, 2.36)	99	2^22^, ^35^	0	NO	NO	NO
TNF‐α	2	REM	0.13 (−2.65, 2.91)	98	2^20^, ^44^	0	NO	NO	NO
e)									
Transfer rate	2	FEM	0.55 (0.29, 1.03)	0	2^15^, ^41^	0	NO	NO	NO
TTP	3	REM	1.33 (0.05, 2.60)	95	1^41^	2	FEM	0.64 (0.34, 0.94)	0

Abbreviations: CI, confidence interval; FEM, fixed‐effects model; ORs, odds ratios; Over or Under, over or underestimated trial which the result had significant difference and was beneficial to TCMs use; Poor trial (Poor) that had at least one domain being considered as high risk of bias; SM, statistical method; SMD, standardized mean difference.

### 3.12 The effects of multi‐ingredient TCM in the oral administration group

The multi‐ingredient TCM formulae had similarity in their main ingredients and functional approximation. In order to identify the most comparable subgroups of studies and potential synergistic effects, a series of planned sensitivity analyses were made. Only the TCMs with significant ORR results have been reported in our analyses. In Table 6, all significant RR results (excluding those with heterogeneity >30%) were ranked in order according to descending RR.

**TABLE 6:** Effects of specific orally administered TCMs on tumor response: single TCMs and combinations

Level	Traditional Chinese medicine	RR	95% CI	No. of Studies, References	No. Part	*I* ^2^ (%)
1	Yiyiren	1.5682	1.2038, 2.0428	3^24^, ^30^, ^38^	288	0.0
1	Fuling	1.5195	1.1890, 1.9419	4^27^, ^30^, ^38^, ^39^	298	0.0
1	Gancao	1.5195	1.1890, 1.9419	4^27^, ^30^, ^38^, ^39^	262	0.0
1	Baizhu	1.4720	1.1948, 1.8136	8^18^, ^24^, ^28^, ^30^, ^32^, ^38^, ^39^, ^40^	600	0.0
1	Sheshecao	1.4705	1.0849, 1.9932	3^24^, ^28^, ^30^	226	0.0
1	Dangshen	1.4602	1.1655, 1.8294	5^28^, ^30^, ^37^, ^38^, ^39^	307	0.0
1	Banxia	1.4324	1.0436, 1.9661	3^18^, ^28^, ^30^	200	0.0
1	Nvzhenzi	1.3954	1.0500, 1.8545	3^30^, ^37^, ^43^	189	0.0
1	Huangqi	1.3767	1.1082, 1.7103	8^18^, ^24^, ^28^, ^30^, ^32^, ^37^, ^40^, ^43^	551	0.0
1	Taizishen	1.3636	0.8420, 2.2086	3^18^, ^32^, ^40^	226	0.0
1	Jixueteng	1.3439	0.9411, 1.9189	4^24^, ^32^, ^37^, ^40^	287	0.0
2	Baizhu+yiyiren	1.5682	1.2038, 2.0428	3^24^, ^30^, ^38^	258	0.0
2	Baizhu+dangshen	1.5195	1.1890, 1.9419	4^28^, ^30^, ^38^, ^39^	278	0.0
2	Baizhu+fuling	1.5195	1.1890, 1.9419	4^28^, ^30^, ^38^, ^39^	278	0.0
2	Baizhu+gancao	1.5195	1.1890, 1.9419	4^28^, ^30^, ^38^, ^39^	278	0.0
2	Dangshen+gancao	1.5195	1.1890, 1.9419	4^28^, ^30^, ^38^, ^39^	278	0.0
2	Fuling+gancao	1.5195	1.1890, 1.9419	4^28^, ^30^, ^38^, ^39^	298	0.0
2	Huangqi+sheshecao	1.4705	1.0849, 1.9932	3^24^, ^28^, ^30^	216	0.0
2	Baizhu+sheshecao	1.4705	1.0849, 1.9932	3^24^, ^28^, ^30^	216	0.0
2	Huangqi+banxia	1.4324	1.0436, 1.9661	3^18^, ^28^, ^30^	200	0.0
2	Huangqi+baizhu	1.4324	1.1048, 1.8572	6^18^, ^24^, ^28^, ^30^, ^32^, ^40^	442	0.0
2	Banxia+baizhu	1.4324	1.0436, 1.9661	3^18^, ^28^, ^30^	200	0.0
2	Baizhu+jixueteng	1.4324	0.9202, 2.2296	3^24^, ^32^, ^40^	242	0.0
2	Huangqi+nvzhenzi	1.3954	1.0500, 1.8545	3^30^, ^37^, ^43^	189	0.0
2	Huangqi+dangshen	1.3902	1.0350, 1.8672	3^28^, ^30^, ^37^	185	0.0
2	Baizhu+taizishen	1.3636	0.8420, 2.2086	3^18^, ^32^, ^40^	226	0.0
2	Huangqi+taizishen	1.3636	0.8420, 2.2086	3^18^, ^32^, ^40^	226	0.0
2	Huangqi+jixueteng	1.3439	0.9411, 1.9189	4^24^, ^32^, ^37^, ^40^	287	0.0
3	Dangshen+fuling+gancao	1.5195	1.1890, 1.9419	4^28^, ^30^, ^38^, ^39^	298	0.0
3	Huangqi+banxia+baizhu	1.4324	1.0436, 1.9661	3^18^, ^28^, ^30^	200	0.0
7	h + b + b + s + d + f + gancao	1.4828	1.0506, 2.0927	2^28^, ^30^	140	0.0

Abbreviations: 95% CI, 95% confidence interval; *I* ^2^%, measure of heterogeneity; RR, risk ratio for tumor response; No. Part., number of participants; 7.h + b + b + b + d + f(huangqi+banxia+baizhu+ sheshecao+dangshen+fuling).

*Level 1: Single TCM*. Sixty ingredients in the formulae have been included in this review. Among them, there are 11 ingredients that have been used in three or more formulae. The Chinese name in pin yin of each ingredient was used to represent the TCMs. According to their frequency in the formulae, TCMs were listed as follows: Huangqi (*n* = 8), baizhu (*n* = 8), dangshen (*n* = 5), jixueteng (n = 4), fuling (*n* = 4), gancao (*n* = 4), yiyiren (*n* = 3), sheshecao (*n* = 3), banxia (*n* = 3), taizishen (*n* = 3), and nvzhenzi (*n* = 3). Then, the RR values were calculated, which are listed in descending order in Table 2. The pooled RR values were divided into two groups. The RR values in the first group were equal to or greater than the total pool. In the second group, the RR values were less than the total pool.

The first group included eight TCMs: yiyiren (*n* = 3), fuling (*n* = 4), gancao (*n* = 4), baizhu (*n* = 8), sheshecao (*n* = 3), dangshen (*n* = 5), banxia (*n* = 3), and nvzhenzi (*n* = 3). In the second group there were only three TCMs, huangqi (RR, 1.3767 [1.1082, 1.7103]), taizishen (RR, 1.3636 [0.8420, 2.2086]), and jixueteng (RR, 1.3439 [0.9411, 1.9189]), which had a lower value than the total pool (RR, 1.3881 [1.1932, 1.6148]) (Table 6).

*Level 2: Combinations of two TCMs*. Compared with the total pool, at this level, the RR values of 14 pairs including baizhu+yiyiren (*n* = 3), baizhu+dangshen(*n* = 4), baizhu+fuling (*n* = 4), baizhu+gancao(*n* = 4), dangshen+gancao (*n* = 4), fuling+gancao (*n* = 4), huangqi+sheshecao (*n* = 3), baizhu+sheshecao (*n* = 3), huangqi+banxia (*n* = 3), huangqi+baizhu (*n* = 6), banxia+baizhu (*n* = 3), baizhu+jixueteng (*n* = 3), huangqi+nvzhenzi (*n* = 3), huangqi+dangshen (*n* = 3) were equal to or greater. Three pairs were lower than the total pool (baizhu+taizishen, huangqi+taizishen, huangqi+jixueteng) (Table 6).

*Level 3: Combinations of 3 TCMs*. At this level, there were two significant pairs from level 2 that were combined with other TCMs that showed significant RRs compared with single TCM group. At this level, the RR values of all pairs including dangshen+fuling+gancao (*n* = 4), huangqi+banxia+baizhu (*n* = 3) were greater than the total pool (Table 6).

*Levels 4 to 7: Combinations of 4 to 7 TCMs*. There were no combinations of 4, 5, 6 TCMs, and there was one combination of 7 which showed an RR equal to the pool: huangqi+banxia+baizhu+sheshecao+dangshen+fuling+gancao (*n* = 2) (Table 6).

### 3.13 TCMs potential synergistic effects selection

Compared with TCM alone, 10 TCM pairs showed higher RR values and potential synergistic effects in group 1, including baizhu+yiyiren (*n* = 3), baizhu+dangshen (*n* = 4), baizhu+fuling (n = 4), baizhu+gancao (n = 4), dangshen+gancao (*n* = 4), fuling+gancao (*n* = 4), huangqi+sheshecao (*n* = 3), baizhu+sheshecao (*n* = 3), huangqi+banxia (*n* = 3), huangqi+nvzhenzi (*n* = 3), while the RR values of two combinations in levels 3–7 with were lower than the level 1, such as huangqi+banxia+baizhu (*n* = 3), huangqi+banxia+baizhu+sheshecao+ dangshen+fuling+gancao (*n* = 2). In all levels, dangshen, fuling, and gancao showed significant ORRs equal or higher than the totalpool at each level.

## 4 DISCUSSION

TCM's essential components are being studied constantly, and more and more research has proven that TCM may assist with tumor treatment.^55^, ^56^ Capecitabine, a 5‐FU prodrug, is an effective first‐line therapy for CRC due to its ease of use and low frequency of ADRs.^57^ Though oxaliplatin‐ or 5‐FU‐based chemotherapy combined with TCM was shown to be more effective than TCM alone in two studies,^58^, ^59^ the efficiency of capecitabine‐based chemotherapy combined with TCM in CRC is yet unknown.

Thirty‐nine studies including 2751 patients were included in meta‐analyses to evaluate the therapeutic CRC regimen capitabine‐based coupled with TCMs clinical effectiveness and ADRs. As a consequence, capecitabine‐based chemotherapy regimens were shown to be more effective when combined with TCM. The ORR and DCR of the oral TCM or non‐oral group (e.g., injection, enema) were shown to be substantially greater than those utilizing capitabine alone, as a consequence of which we exhibited. Improving immunological function and overall well‐being is critical for cancer patients undergoing treatment. We compared the QOL and the number of peripheral blood lymphocytes in each group as part of our research. According to the findings, combining capecitabine‐based chemotherapy with TCM improved CD3^+^ T cells, CD4^+^ T cells, CD4^+^/CD8^+^ T cells ratio, and NK cells, as well as overall QOL. In addition, it has the potential to decrease tumor marker expression levels (CEA, CA199, and CA125). This will assist the patient's immune system, allowing him or her to fight off tumor recurrence and metastasis in the future. T‐lymphocyte expression is linked to poor prognosis and tumor metastasis,^60^ such as CD3^+,^ ^61^ CD4^+,^ ^62^ CD4^+^/CD8^+^ T cell ratio,^63^ and NK cells,^64^ increasing immune function to inhibit tumor growth.^65^ An important clinical biomarker for gastrointestinal malignancies is a cell surface glycoprotein called carcinoembryonic antigen (CEA)^66^. There has been an increase in CEA overexpression in 90% of gastrointestinal cancers, including CRC tumor recurrence is predicted by an increase in postoperative CA125 and CA199 levels, and this information is critical for the diagnosis of digestive system cancer.^67^, ^68^

TCMs used orally or intravenously showed promise in the treatment of CRC. Specific plant‐based TCMs were further analyzed and shown to have substantially greater contributions to the RR value, including yiyiren, fuling, gancao, baizhu, sheshecao, dangshen, banxia, and nvzhenzi. Dangshen, fuling, and gancao all contributed considerably more to the RR value than the others at all levels. Capecitabine‐based chemotherapy for CRC may benefit from the addition of TCMs.

## 5 CONCLUSION

Our research found that the combination of TCM and capecitabine‐based chemotherapy was more effective than the capecitabine‐only regimen. Additionally, it has the potential to decrease adverse responses in patients, enhance survival rates, and the body's capacity to fight off infection, lower tumor marker expression levels, and even slow tumor development. Specific TCMs may have the potential to improve the efficacy of capecitabine‐based chemotherapy for CRC.

## AUTHOR CONTRIBUTIONS

Hui‐Zhong Jiang and Ya‐Li Jiang conceived of and designed the study. They had full access to all data in the study and took responsibility for the integrity of the data, the accuracy of the data analysis, and the writing of the report. Yang Bing, Feng‐Xi Long, Zhu Yang, and Dong‐Xin Tang critically revised the report. Hui‐Zhong Jiang and Ya‐Li Jiang performed the statistical analyses. All the authors contributed to the data acquisition and analyses. The authors have reviewed and approved the final version of the manuscript.

## CONFLICT OF INTEREST

The authors declare that they have no competing interests.

## Supporting information


Figure S1
Figure S2;
Figure S3;
Figures S4‐S5

Appendix S1

## Data Availability

Data sharing is not applicable to this article as no new data were created or analyzed in this study.

## References

[1] Dekker E , Tanis PJ , Vleugels JLA , Kasi PM , Wallace MB . Colorectal cancer. Lancet. 2019;394:1467‐1480. 31631858 10.1016/S0140-6736(19)32319-0

[2] Bray F , Ferlay J , Soerjomataram I , Siegel RL , Torre LA , Jemal A . Global cancer statistics 2018: GLOBOCAN estimates of incidence and mortality worldwide for 36 cancers in 185 countries. CA Cancer J Clin. 2018;68(6):394‐424. 30207593 10.3322/caac.21492

[3] Messersmith WA . NCCN guidelines updates: management of metastatic colorectal cancer. J Natl Compr Canc Netw. 2019;17:599‐601. 31117039 10.6004/jnccn.2019.5014

[4] Brown KGM , Solomon MJ , Mahon K , O’Shannassy S . Management of colorectal cancer. BMJ. 2019;366:l4561. 31439545 10.1136/bmj.l4561

[5] Benson AB , Venook AP , Al‐Hawary MM , et al. Version 2.2018, NCCN clinical practice guidelines in oncology. J Natl Compr Canc Netw. 2018;16(7):874–901. 30006429 10.6004/jnccn.2018.0061 PMC10203817

[6] Xie YH , Chen YX , Fang JY . Comprehensive review of targeted therapy for colorectal cancer. Signal Transduct Target Ther. 2020;5(1):22 Published 2020 Mar 20. 32296018 10.1038/s41392-020-0116-z PMC7082344

[7] Suzuki K , Takaharu K , Muto Y , et al. XELIRI regimen plus continuous treatment with bevacizumab is well‐tolerated and effective in metastatic colorectal cancer patients in a second‐line setting involving the sequential administration of XELOX and XELIRI. Mol Clin Oncol. 2014;2(5):827‐832. 25054053 10.3892/mco.2014.306 PMC4106724

[8] Kadoyama K , Miki I , Tamura T , Brown JB , Sakaeda T , Okuno Y . Adverse event profiles of 5‐fluorouracil and capecitabine: data mining of the public version of the FDA adverse event reporting system, AERS, and reproducibility of clinical observations. Int J Med Sci. 2012;9(1):33‐39. 22211087 10.7150/ijms.9.33 PMC3222088

[9] Kong MY , Li LY , Lou YM , Chi HY , Wu JJ . Chinese herbal medicines for prevention and treatment of colorectal cancer: from molecular mechanisms to potential clinical applications. J Integr Med. 2020;18(5):369‐384. 32758397 10.1016/j.joim.2020.07.005

[10] Wang Y , Liu P , Fang Y , et al. The effect of long‐term traditional Chinese medicine treatment on survival time of colorectal cancer based on propensity score matching: a retrospective cohort study. Evid Based Complement Alternat Med. 2020;31(2020):7023420. 10.1155/2020/7023420 PMC7013320 32089727

[11] Sun Q , He M , Zhang M , et al. Traditional Chinese medicine and colorectal cancer: implications for drug discovery. Front Pharmacol. 2021;12:685002. Published 2021 Jul 1. 34276374 10.3389/fphar.2021.685002 PMC8281679

[12] Moher D , Liberati A , Tetzlaff J , et al. Preferred reporting items for systematic reviews and meta‐analyses: the PRISMA statement. Ann Intern Med. 2009;151:264‐269. 19622511 10.7326/0003-4819-151-4-200908180-00135

[13] Friedrich JO , Adhikari NK , Beyene J . Inclusion of zero total event trials in meta‐analyses maintains analytic consistency and incorporates all available data. BMC Med Res Methodol. 2007;7:5. 17244367 10.1186/1471-2288-7-5 PMC1783664

[14] Zhao Y . Clinical observation on Changing drink combined with chemotherapy in the treatment of rectal cancer. Guangming J Chin Med. 2017;32(12):1790‐1791.

[15] Shi XY , Zhang J , Meng W , et al. Clinical observation and immune regulation effect of erling Yiren decoction combined with cantharis acid sodium vitamin B6 in treatment of advanced colon cancer. Liaoning J Trad Chin Med. 2018;45(5):987‐990.

[16] Chen WJ , He JS , Lv XP . The clinical effect of Lingling Yiren soup and conventional western medicine in the treatment of colon cancer and its influence on cellular immune function status and serological indicators. Zhejiang J Tradit Chin Med. 2019;54(09):664‐665.

[17] Gu G . Efficacy of the Erling Yiren decoction plus conventional Western medicine on the advanced colon cancer with cellular immune function and serum indexes. Clin J Chin Med. 2021;13(1):13‐15.

[18] Zhang WW , Chen J , Xie GQ , et al. Clinical study of spleen and detoxification combined with cappeitalin tablets in the treatment of advanced colorectal cancer. Acad J Shanghai Univ Tradit Chin Med. 2013;27(04):31‐34.

[19] Cui HJ . Clinical study of Jianpi Quyu decoction with chemotherapy in the treatment of patients with advanced colorectal tumor. J Pract Tradit Chin Int Med. 2011;25(8):48‐50.

[20] Sun F , Chen H , Hu FL , et al. Clinical observation of the effect of spleen and Yin Yin soup on XELOX chemotherapy immunity of rectal cancer. J New Chin Med. 2012;44(7):65‐66.

[21] Ding P , Lu ZW , Wei WM , et al. Clinical observation of Kang’ ai injection combined with capecitabine and oxaliplatin scheme in the treatment of advanced colon cancer. Shaanxi J Tradit Chin Med. 2017;3:351‐352.

[22] Min GL , Chen HL . Effect of Shiyiwei Shenqi capsules combined with XELOX regimen in treatment of advanced colorectal cancer. Chin Arch Tradit Chin Med. 2020;38(07):220‐223.

[23] Pan W , Wu GX , Wang WJ . Effect of Shiling dispersing flavor and XELOX chemotherapy on cellular immune function and quality of survival in patients with spleen deficiency and humid heat syndrome after colorectal cancer. Mod J Integr Tradit Chin West Med. 2019;28(24):2690‐2693.

[24] Yue DM . The effect of XELIRI regimen with tongtai decoction in patients with advanced colorectal cancer. Mod Digest Intervent. 2015;20(06):596‐598.

[25] Zhou J , Wen QX , Xu B . Effect analysis of traditional Chinese medicine treatment in advanced colorectal cancer maintenance period. Chin J Woman Child Health Res. 2016;27(S1):255.

[26] Gu N , Li ZG . Xiaoaiping injection combined with XELOX chemotherapy for colorectal cancer. J Pract Oncol. 2017;32(02):172‐175.

[27] Xiao WY . Clinical observation of replenishing qi and spleen method and XELOX scheme in the treatment of advanced colorectal cancer. Pract J Cancer. 2013;28(03):305‐306.

[28] Bin XY . Clinical observation of colon cancer treated with Zhenxiang capsules and capecitabine. World J Integr Tradit Western Med. 2012;7(06):492–493.

[29] Yao C , Ren HC . Effect of 21 cases of integrated traditional Chinese and western medicine on advanced colorectal cancer. J New Chin Med. 2014;46(12):161‐163.

[30] Guo YL , Zhang ZX , Liu GQ . Effect of integrated traditional Chinese and western medicine on advanced colon cancer. J Huaihai Med. 2018;36(04):461‐462.

[31] Chen WH , Tian Y , Shi YM , et al. Observation on the efficacy of integrated traditional Chinese and western medicine on advanced colon cancer. Mod J Integr Tradit Chin Western Med. 2014;23(11):1172‐1174.

[32] Liu JH , Yang HJ , Liu DW , et al. Effects of traditional Chinese medicine prescription agent on immune function and bone marrow suppression in patients with colorectal cancer chemotherapy. Chin J Public Health Eng. 2020;19(02):294‐296.

[33] Yao Q , Zhang YL , Deng JL , et al. Clinical observation of TCM and XELOX treatment of advanced colorectal cancer. Mod J Integr Tradit Chin Western Med. 2017;26(30):3399‐3401.

[34] Li AH , Jiang Y , Guo F , et al. Effect of traditional Chinese medicine enema and Xeloda in the treatment of advanced colorectal cancer. Diet Health Care. 2020;007(005):127‐128.

[35] Zhou LY , You JL , Shan ZZ , et al. Clinical study of applying three‐step cycle therapy of Chinese herbal medicine and chemotherapy in the treatment of stage IV colonic cancer. J Sichuan Tradit Chin Med. 2016;34(12):90‐93.

[36] Jiao SJ , Fan CQ , An GY , et al. Study on nourishing soup combined with capecitabine in advanced colorectal cancer with syndrome of deficiency of both Qi and blood after first‐line chemotherapy. Mod J Integr Tradit Chin Western Med. 2016;25(12):1258–1260.

[37] Liu Q . Clinical observation of Fuzheng Shengbai oral liquid combined with XELOX schedule in the treatment of advanced colorectal cancer. Clin J Tradit Chin Med. 2018;30(9):1688‐1690.

[38] Dong J , Lu N , Shi GJ . Clinical study on patients with advanced liver metastasis from colon cancer treated with Fufang Tengligen preparation combined with XELIRI chemotherapy regimen Chin J Tradit Med Sci Technol. 2017;24(02):132–134.

[39] Sun YF , You JL . Efficacy of Peiben Yiyang soup and CapeOx chemotherapy regimen in the treatment of peritoneal metastasis of spleen, kidney and Yang deficiency rectal cancer. Beijing J Tradit Chin Med. 2019;38(8):799‐801.

[40] Xu WR , Zhang Q , Fu Q , et al. Influence of Shengxue decoction on myelosuppression and immune function of metastatic colorectal cancer patients after chemotherapy. China J Tradit Chin Med Pharm. 2015;30(06):2230‐2232.

[41] Xu YF . Clinical research on reducing recurrence and metastasis of II,III period colorectal cancer in the integrative medicine. Clin J Chin Med. 2016;8(11):99‐100.

[42] Ding JF . Effects of Eddie injection on the quality of life in patients after rectal cancer. J New Chin Med. 2015;47(10):176‐178.

[43] Xie JB , Jia CH , Yuan Y , et al. Clinical efficacy of Boerning capsules plus XELOX regimen in treatment of colorectal cancer. World Chin J Digestol. 2017;25(12):1110‐1114.

[44] Ma T , Yang D , Chu J , et al. Clinical efficacy of Buqi Yichang decoction in the treatment of postoperative patients with colorectal cancer and its influence on immune function. World J Integr Tradit West Med,2020,15(12):2161–2165+2172.

[45] Bian SC , Hong L , Wan HJ . Solid ben tumor suppression combined with carbapitabin for patients and effects on immune function. Jiangxi J Tradit Chin Med. 2013;44(4):30‐32.

[46] Li Y , Liu H , Zhang YB , et al. Effect of Jianpi Fuzheng recipe on T‐cell Subsets,Treg cells and quality of life in patients with colorectal Cancer afer operation chemotherapy. Guiding J Tradit Chin Med Pharm,2019,25(6):49–52+59.

[47] Xie J , Tang W . Effect of spleen spleen and qi method on postoperative complications, immune function and quality of life in patients with colorectal cancer. Henan Tradit Chin Med. 2017;37(2):273‐275.

[48] Qi J . Clinical observation of Ningchangyin combined with oxaliplatin and capecitabine in the treatment of patients with colon cancer. Guide China Med. 2018;16(31):14‐15.

[49] Zhou M , Zhu P , Li T . The effect of Sanmiao granule flushing on adjuvant chemotherapy after colorectal cancer was observed. Inner Mongolia J Tradit Chin Med. 2017;36(15):9‐10.

[50] Wu Y , Sha LL . Clinical observation on Prunella vulgaris tablets combined with routine chemotherapy in improving postoperative prognosis of patients with rectal cancer. J Hubei Univ Chin Med. 2016;18(5):25‐28.

[51] Xiao C , Cao B , Wu X , et al. Effect of tonifying Qi and nourishing Yin prescription for reducing phlegm on CEA and CA50 in postoperative patients with colorectal cancer. Acta Med Mediterr. 2020;36(1):711‐716.

[52] Xiao C , Cao B , Wu X , et al. Effect of Yiqi Yangyin Huatan recipe combined with xelox chemotherapy on immune function in patients with colorectal cancer surgery. Acta Med Mediterr. 2020;36(6):3729‐3736.

[53] Miller AB , Hoogstraten B , Staquet M , Winkler A . Reporting results of cancer treatment. Cancer. 1981;47(1):207‐214. 7459811 10.1002/1097-0142(19810101)47:1<207::aid-cncr2820470134>3.0.co;2-6

[54] Watanabe H , Yamamoto S , Kunitoh H , et al. Tumor response to chemotherapy: the validity and reproducibility of RECIST guidelines in NSCLC patients. Cancer Sci. 2003;94(11):1015‐1020. 14611681 10.1111/j.1349-7006.2003.tb01394.x PMC11160149

[55] Qi F , Zhao L , Zhou A , et al. The advantages of using traditional Chinese medicine as an adjunctive therapy in the whole course of cancer treatment instead of only terminal stage of cancer. Biosci Trends. 2015;9:16‐34. 25787906 10.5582/bst.2015.01019

[56] Chien TJ , Liu CY , Lu RH , Kuo CW , Lin YC , Hsu CH . Therapeutic efficacy of traditional Chinese medicine, “Kuan‐sin‐Yin”, in patients undergoing chemotherapy for advanced colon cancer—a controlled trial. Complement Ther Med. 2016;29:204‐212. 27912948 10.1016/j.ctim.2016.10.001

[57] García‐Alfonso P , Muñoz Martín AJ , Ortega Morán L , Soto Alsar J , Torres Pérez‐Solero G , Blanco Codesido M , Calvo Ferrandiz PA , Grasso Cicala S Oral drugs in the treatment of metastatic colorectal cancer. Ther Adv Med Oncol. 2021;13:17588359211009001. 10.1177/17588359211009001 PMC8111515 33995592

[58] Chen M , May BH , Zhou IW , Xue CCL , Zhang AL . Metaanalysis of oxaliplatin‐based chemotherapy combined with traditional medicines for colorectal cancer: contributions of specific plants to tumor response. Integr Cancer Ther. 2016;15:40‐59. 26254190 10.1177/1534735415596424 PMC5736077

[59] Chen P , Ni W , Xie T , Sui X . Meta‐analysis of 5‐fluorouracil‐based chemotherapy combined with traditional Chinese medicines for colorectal cancer treatment. Integr Cancer Ther. 2019;18:1534735419828824. 10.1177/1534735419828824 PMC7242800 30791729

[60] Stenström J , Hedenfalk I , Hagerling C . Regulatory T lymphocyte infiltration in metastatic breast cancer‐an independent prognostic factor that changes with tumor progression. Breast Cancer Res. 2021;23(1):27. 33602289 10.1186/s13058-021-01403-0 PMC7893927

[61] Gooden MJ , de Bock GH , Leffers N , et al. The prognostic influence of tumour‐infiltrating lymphocytes in cancer: a systematic review with meta‐analysis. Br J Cancer. 2011;105(1):93‐103. 21629244 10.1038/bjc.2011.189 PMC3137407

[62] Marty Pyke R , Thompson WK , Salem RM , Font‐Burgada J , Zanetti M , Carter H . Evolutionary pressure against MHC class II binding cancer mutations. Cell. 2018;175(2):416‐428.e13. 30245014 10.1016/j.cell.2018.08.048 PMC6482006

[63] Jie HY , Ye JL , Zhou HH , Li YX . Perioperative restricted fluid therapy preserves immunological function in patients with colorectal cancer. World J Gastroenterol. 2014;20(42):15852‐15859. 25400472 10.3748/wjg.v20.i42.15852 PMC4229553

[64] Dogra P , Rancan C , Ma W , et al. Tissue determinants of human NK cell development, function, and residence. Cell. 2020;180(4):749‐763.e13. 32059780 10.1016/j.cell.2020.01.022 PMC7194029

[65] Li L , Goedegebuure SP , Gillanders WE . Preclinical and clinical development of neoantigen vaccines. Ann Oncol. 2017;28:xii11‐xii17. 29253113 10.1093/annonc/mdx681 PMC5834106

[66] Wang N , Patel H , Schneider IC , Kai X , Varshney AK , Zhou L . An optimal antitumor response by a novel CEA/CD3 bispecific antibody for colorectal cancers. Antib Ther. 2021;4(2):90‐100. 34169228 10.1093/abt/tbab009 PMC8220303

[67] Zhang D , Hou W , Liu F , et al. Metformin reduces serum CA199 levels in type 2 diabetes Chinese patients with time‐effect and gender difference. Diabetes Technol Ther. 2015;17(2):72‐79. 25548963 10.1089/dia.2014.0176 PMC4321771

[68] Kalogera E , Scholler N , Powless C , et al. Correlation of serum HE4 with tumor size and myometrial invasion in endometrial cancer. Gynecol Oncol. 2012;124(2):270‐275. 22037318 10.1016/j.ygyno.2011.10.025 PMC3913473

