# Supplementary material for: Traditional Chinese medicines and capecitabine‐based chemotherapy for colorectal cancer treatment: A meta‐analysis
Source: Cancer Med. 2022 Jun 1;12(1):236–55. doi: 10.1002/cam4.4896 (PMC9844598; doi:10.1002/cam4.4896)
Supplement: Supplementary file 1 — Figure S1 Figure S2; Figure S3; Figures S4‐S5 [file CAM4-12-236-s001.pdf]

Supplementary Materials

Traditional Chinese Medicines and Capecitabine-Based Chemotherapy for Colorectal Cancer Treatment：A Meta-Analysis

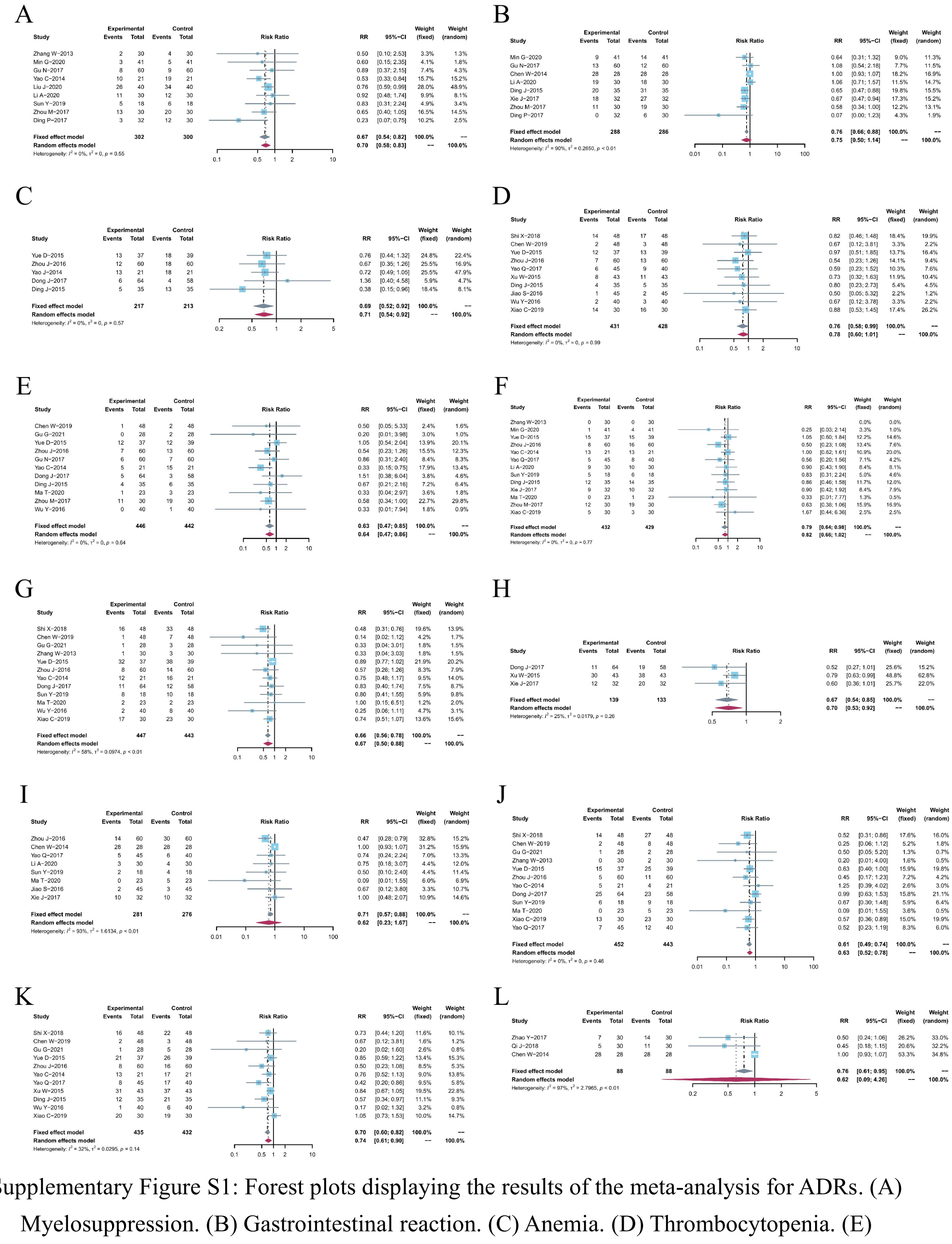

Supplementary Figure S1: Forest plots displaying the results of the meta-analysis for ADRs. (A) Myelosuppression. (B) Gastrointestinal reaction. (C) Anemia. (D) Thrombocytopenia. (E) Liver/Renal dysfunction. (F) Neurotoxicity. (G) Nausea/vomiting. (H) Neutropenia. (I) Hand-foot syndrome. (J) Diarrhoea. (K) Leukopenia. (L) Hematological toxicity.

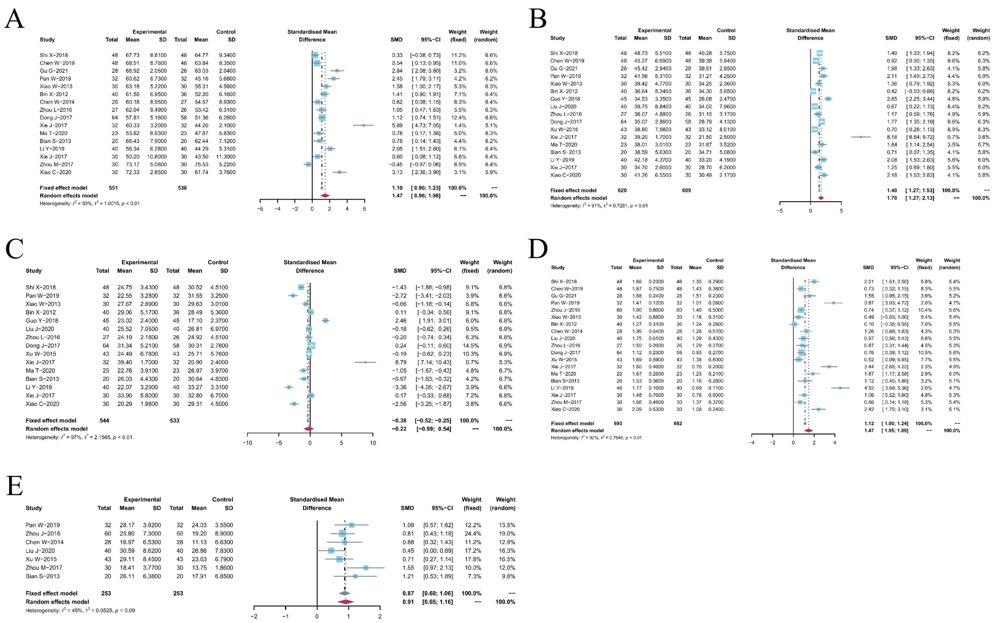

Supplementary Figure S2: Forest plots displaying the results of the meta-analysis for the levels of peripheral blood lymphocytes. (A) CD3^{+} T cells. (B) CD4^{+} T cells. (C) CD8^{+} T cells. (D) CD4^{+}/CD8^{+} T Cells ratio. (E) NK cells.

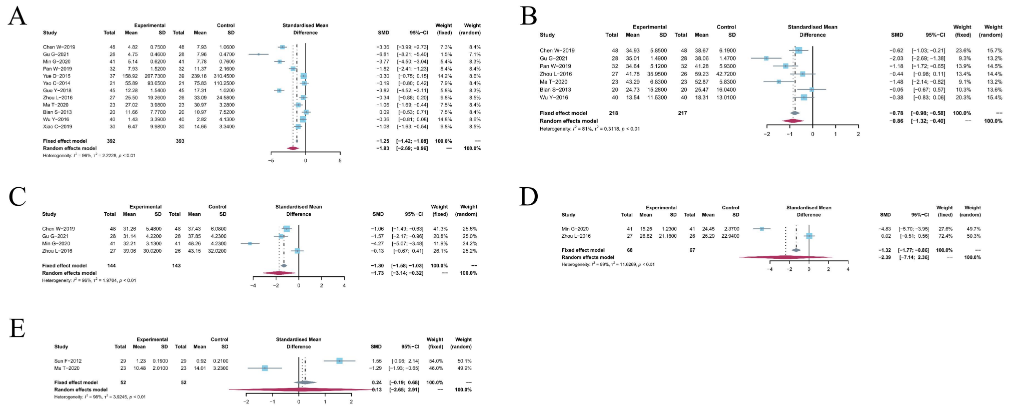

Supplementary Figure S3: Forest plots displaying the results of the meta-analysis for the tumor markers and related factors. (A) CEA. (B) CA199. (C) CA125. (D) CA724. (E) TNF-α.

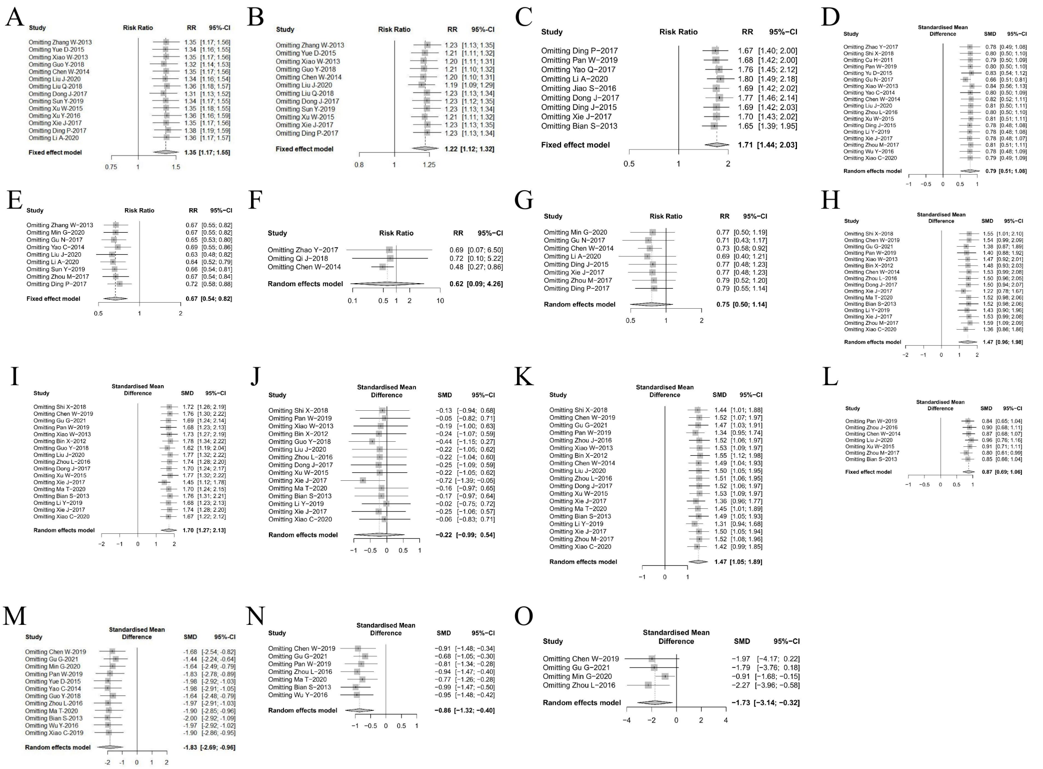

Supplementary Figure S4: Meta-analysis results of Sensitivity analysis(1). (A) ORR. (B) DCR. (C) KPS (number). (D) KPS (mean±SD). (E) Myelosuppression. (F) Hematological toxicity. (G) Gastrointestinal reaction. (H) CD3^{+} T cells. (I) CD4^{+} T cells. (J) CD8^{+} T cells. (K) CD4^{+}/CD8^{+} T Cells ratio. (L) NK cells. (M) CEA. (N) CA199. (O) CA125. (P) CA724. (Q) TNF-α. (R) 1-year Survival rate. (S) 2-year Survival rate. (T) transfer rate. (U) TTP.

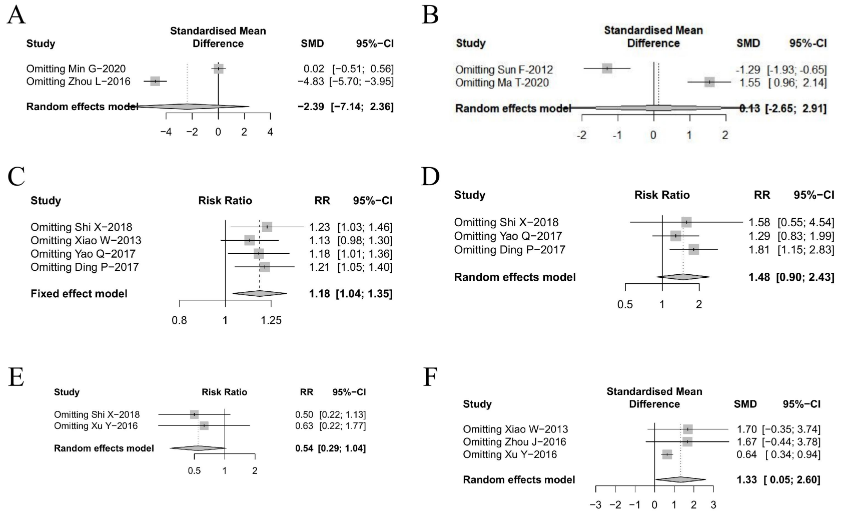

Supplementary Figure S5: Meta-analysis results of Sensitivity analysis(2). (A) CA724. (B) TNF-α. (C) 1-year Survival rate. (D) 2-year Survival rate. (E) transfer rate. (F) TTP.
