# Supplementary material for: Traditional Chinese medicines and capecitabine‐based chemotherapy for colorectal cancer treatment: A meta‐analysis
Source: Cancer Med. 2022 Jun 1;12(1):236–55. doi: 10.1002/cam4.4896 (PMC9844598; doi:10.1002/cam4.4896)
Supplement: Supplementary file 2 — Appendix S1 [file CAM4-12-236-s002.pdf]

International Platform of Registered Systematic Review and Meta-analysis Protocols

INPLASY PROTOCOL

To cite: Jiang et al. Meta-Analysis of Capecitabine-Based Chemotherapy Combined With Traditional Chinese Medicines for Colorectal Cancer Treatment. Inplasy protocol 202130095. doi: 10.37766/inplasy2021.3.0095

Received: 25 March 2021

Published: 25 March 2021

Corresponding author:
Dongxin Tang

tangdongxin@sina.com

Author Affiliation:
Guizhou University of Traditional Chinese Medicine

Support: **NSFC (NO.81760814).**

Review Stage at time of this submission: **The review has not yet started.**

Conflicts of interest:
None declared.

Meta-Analysis of Capecitabine-Based Chemotherapy Combined With Traditional Chinese Medicines for Colorectal Cancer Treatment

Jiang, HZ[1]; Jiang, YL[2]; Yu, D[3]; Long, FX[4]; Luo, L[5]; Yang, Z[6]; Tang, DX[7].

Review question / Objective: **The aim of this systematic review and meta-analysis of randomized controlled trials is to evaluate the efficacy and safety of Traditional Chinese Medicines combined with Capecitabine-Based chemotherapy for Colorectal Cancer.**
Condition being studied: **Capecitabine-Based Chemotherapy Combined With Traditional Chinese Medicines for Colorectal Cancer Treatment.**
Information sources: **The Eight electronic databases including Cochrane Library, PubMed, Web of Science (WOS), Excerpt Medica Database (Embase), Chinese Biomedical Literature Database (CBM), China National Knowledge Infrastructure (CNKI), China Scientific Journal Database (VIP) and Wanfang Database will be systematically searched for eligible studies from their inception to March 2021. In addition, the reference list from original reports and previous reviews will be reviewed, and manually selected for other available publications. Language is limited with English and Chinese. When the information is incomplete, we will try to connect with the authors.**

INPLASY registration number: This protocol was registered with the International Platform of Registered Systematic Review and Meta-Analysis Protocols (INPLASY) on 25 March 2021 and was last updated on 25 March 2021 (registration number INPLASY202130095).

INTRODUCTION

Review question / Objective: **The aim of this systematic review and meta-analysis of randomized controlled trials is to evaluate the efficacy and safety of Traditional Chinese Medicines combined with Capecitabine-Based chemotherapy for Colorectal Cancer.**

Condition being studied: **Capecitabine-Based Chemotherapy Combined With**

Jiang et al. Inplasy protocol 202130095. doi:10.37766/inplasy2021.3.0095 Downloaded from https://inplasy.com/inplasy-2021-3-0095/

Traditional Chinese Medicines for Colorectal Cancer Treatment.

METHODS

Participant or population: All included cases must be confirmed as colorectal cancer by histopathological examination. There will be no restriction on gender, race, or nation. Patients with non primary colorectal cancer or other tumors were excluded.

Intervention: The RCTs that used traditional Chinese medicine combined with Capecitabine-Based Chemotherapy will be included. There will be no restrictions on the types of traditional Chinese medicine.

Comparator: In the control group, CRC patient treated with the same conventional treatment as intervention group in the same original study.

Study designs to be included: All available RCTs that investigated the efficacy and safety of TCM-mediated therapy in patients diagnosed with advanced GC will be included.

Eligibility criteria: This study will include randomized controlled trials (RCTs) or quasi-RCTs, and high-quality prospective cohort studies that evaluated the efficacy and safety of Traditional Chinese Medicines combined with Capecitabine-Based chemotherapy for Colorectal Cancer. Articles without sufficient available data, noncomparative studies, non-peer reviewed articles, literature reviews, meta-analysis, case reports and series, meeting abstracts, animal studies, letter to the editor, editorials, commentaries, and other unrelated studies will be all excluded from analysis.

Information sources: The Eight electronic databases including Cochrane Library, PubMed, Web of Science (WOS), Excerpt Medica Database (Embase), Chinese Biomedical Literature Database (CBM), China National Knowledge Infrastructure (CNKI), China Scientific Journal Database (VIP) and Wanfang Database will be systematically searched for eligible studies from their inception to March 2021. In addition, the reference list from original reports and previous reviews will be reviewed, and manually selected for other available publications. Language is limited with English and Chinese. When the information is incomplete, we will try to connect with the authors.

Main outcome(s): The primary outcomes in present analysis included short-term and long-term clinical efficacy, and adverse effects (AEs) according to Organization (WHO) criteria and Response Evaluation Criteria in Solid Tumors (RECIST Criteria). (I) Short-term clinical efficacy: the short-term tumor response included complete response (CR), partial response (PR), stable disease (SD), progressive disease (PD), overall response rate (ORR) and disease control rate (DCR). ORR was defined as the sum of CR and PR, and DCR was the sum of CR, PR and SD. (II) Longterm clinical efficacy: 1-5 year Overall survival (OS, which is defined as the time from the date of randomization to death from any cause); 1-5 year progression free survival (DFS, which is the time from date of random assignment to date of recurrence or death).

Additional outcome(s): Quality of life (QOL), Adverse Drug Reactions (ADRs) and the levels of peripheral blood lymphocytes. QOL is considered to be improved when Karnofsky Performance Status (KPS) score is ten points higher after being treated. ADRs are accessed by measuring hematotoxicity (neutropenia, anemia, thrombocytopenia), gastrointestinal toxicity (nausea and vomiting, diarrhea), hepatic or renal dysfunction, neurotoxicity, alopecia and stomatitis, according to WHO Recommendations for Grading of Acute and Subacute Toxicity or NCI Common Terminology Criteria for Adverse Events (CTCAE). The levels of peripheral blood lymphocytes will be assessed by measuring the T-lymphocyte subsets such as the proportion of CD3+, CD3+CD4+, and CD3+CD8+ T cells; the ratio of CD4+/CD8+ T cells; and the proportion of natural killer cells (NK cells).

Jiang et al. Inplasy protocol 202130095. doi:10.37766/inplasy2021.3.0095 Downloaded from https://inplasy.com/inplasy-2021-3-0095/

Data management: Two reviewers (Huizhong Jiang and Yali Jiang) will be responsible for the data extraction independently according to the Cochrane Handbook for Systematic Reviews of Intervention. The following data will be extracted from eligible literatures: the first author, year of publication, country of study, participants (sample size, TNM stage, age, gender, inclusion and exclusion criteria, etc.), details of all experimental and control interventions regimen (manufacturer of the drugs, dosage of JLC, administration route, duration of treatment, follow-up time, etc.), outcomes (ORR, DCR, OS, DFS, QoL, immune function and adverse effects). For survival outcomes, Hazard ratios (HRs) with corresponding 95% confidence intervals (CIs) will be extracted from trials or be estimated from Kaplan–Meier survival curves by established methods. Any disagreements will be resolved by discussion, and a third reviewer (Dongxin Tang) will make the final decision. Excluded studies and the reasons for exclusion will be listed in a table.

Quality assessment / Risk of bias analysis: The quality of the included RCTs will be assessed independently by 2 investigators(Huizhong Jiang and Yali Jiang) in terms of sequence generation , allocation concealment, blinding, incomplete outcome data, selective reporting and other bias, according to the guidance of the Cochrane Handbook for Systematic Review of Interventions. Evidence quality will be classified as low risk, high risk, or unclear risk of bias in accordance with the criteria of the risk of bias judgment. Any disagreements will be resolved via discussion with a third researcher (Dongxin Tang).

Strategy of data synthesis: Statistical analyses will be performed using Review Manager 5.3 (Nordic Cochran Centre, Copenhagen, Denmark) and Stata 14.0 (Stata Corp., College Station, TX, USA) statistical software. The outcomes were mainly represented by risk ratio (RR) and standardized mean difference (SMD) with its 95% CIs. A two-tailed P value < 0.05 was considered statistically significant. Cochrane's Q-test and I2 statistics were used to assess heterogeneity between studies; P ≤ 0.1 or I2 $> 50\%$ indicates statistical heterogeneity. A fixed effect model will be used to calculate the outcomes when statistical heterogeneity is absent; otherwise, the random effects model was considered according to the DerSimonian and Laird method.

Subgroup analysis: If the data are available and sufficient, subgroup and metaregression analysis will be conducted to explore the source of heterogeneity with respect to age, gender, region, tumor stage, course of treatment and therapeutic regimens.

Sensitivity analysis: Sensitivity analysis was conducted to explore an individual study's influence on the pooled results by deleting one single study each time from pooled analysis. A summary table will report the results of the sensitivity analyses.

Language: Language is limited with English and Chinese.

Country(ies) involved: China.

Other relevant information: Publication bias analysis: We will detect publication biases and poor methodological quality of small studies using funnel plots if 10 or more studies are included in the meta-analysis. Begg's and Egger regression test will be utilized to detect the funnel plot asymmetry. If reporting bias is suspected, we will consult the study author to get more information. If publication bias existed, a trim-and-fill method should be applied to coordinate the estimates from unpublished studies, and the adjusted results were compared with the original pooled RR. Evidence evaluation: The evidence grade will be determined by using the guidelines of the Grading of Recommendations, Assessment, Development, and Evaluation (GRADE). The quality of all evidence will be evaluated as 4 levels (high, moderate, low, and very low).

Jiang et al. Inplasy protocol 202130095. doi:10.37766/inplasy2021.3.0095 Downloaded from https://inplasy.com/inplasy-2021-3-0095/

Keywords: Capecitabine; colorectal cancer; traditional Chinese medicine; meta-analysis.

Dissemination plans: We will disseminate the results of this systematic review by publishing the manuscript in a peer-reviewed journal or presenting the findings at a relevant conference.

Contributions of each author:

Author 1 - Huizhong Jiang.
Email: jianghz93@126.com
Author 2 - Yali Jiang.
Email: jylxtc777@163.com
Author 3 - Dan Yu.
Email: 2689129306@qq.com
Author 4 - Fengxi Long.
Email: 39854265@qq.com
Author 5 - Li Luo.
Email: 904211086@qq.com
Author 6 - Zhu Yang.
Email: yangzhu20150426@163.com
Author 7 - Dongxin Tang.
Email: tangdongxin@sina.com

Jiang et al. Inplasy protocol 202130095. doi:10.37766/inplasy2021.3.0095 Downloaded from https://inplasy.com/inplasy-2021-3-0095/
